# Strategic Governance of Artificial Intelligence–Enabled Clinical Algorithm Development: Formative Evaluation of the Semiautomatic Clinical Algorithm Development Framework

**DOI:** 10.2196/90273

**Published:** 2026-03-12

**Authors:** Sang Hyun Ahn, Junhewk Kim

**Affiliations:** 1 MoDoc AI Inc. Needham, MA United States; 2 Mobile Doctor Inc. Seoul Republic of Korea; 3 Department of Occupational and Environmental Medicine Dankook University Hospital Cheonan Republic of Korea; 4 Department of Dental Education College of Dentistry Yonsei University Seoul Republic of Korea

**Keywords:** AI-enabled uncertainty, strategic governance, digital health management, health care leadership, human-in-the-loop, organizational implementation, clinical algorithm development, human-AI collaboration, decision-making frameworks, learning health systems

## Abstract

**Background:**

Health care leaders face a strategic dilemma: traditional expert-led content development ensures safety but is too slow for digital innovation, whereas artificial intelligence (AI) automation offers speed but introduces risks from hallucinations. Resolving this tension requires governance frameworks that balance operational efficiency with rigorous accountability for patient safety.

**Objective:**

This study describes the development process and conducts a formative evaluation of the Semiautomatic Clinical Algorithm Development (S-ACAD) framework as an industry-driven implementation strategy. We aimed to assess the feasibility of this “human-in-the-loop” governance model in balancing the need for operational efficiency with the rigorous safety standards required for pediatric emergency guidance.

**Methods:**

We conducted a prospective, single-day proof-of-concept case study focusing on pediatric febrile seizures. A single physician expert executed a 4-phase workflow: (1) parallel data collection using multiple AI agents, (2) AI-assisted synthesis, (3) iterative refinement via “AI sparring,” and (4) final clinical validation. The resulting algorithm was reviewed by 2 independent external pediatric specialists. We benchmarked this process against a fully automated system (Fully Autonomous Clinical Algorithm Development [F-ACAD]) to illustrate comparative efficiency and safety trade-offs.

**Results:**

In this single execution, the S-ACAD framework produced a parent-actionable febrile seizure algorithm in approximately 245 minutes. Two independent pediatric specialists (N=2) reviewed the output and did not identify medically inaccurate sections or critical safety errors requiring mandatory correction, and both rated overall clinical validity highly (9.0 and 9.5 out of 10). During the workflow, 19 human expert interventions were recorded, with clinical judgment (n=8, 42.1%) and safety review (n=5, 26.3%) as the most frequent categories in an exploratory post hoc analysis. By comparison, the fully automated approach (F-ACAD) completed the task in approximately 68 minutes, but its own AI critics identified 17 issues (9 high-priority), including concerns related to emergency response clarity and standard-of-care alignment.

**Conclusions:**

These preliminary findings suggest that the S-ACAD framework may offer a potential pathway for “active governance” in AI-assisted clinical content development. In this proof-of-concept case, the framework combined rapid AI-assisted drafting with continuous expert oversight and independent clinical review, suggesting the potential to reduce turnaround time while maintaining safety safeguards. However, these results are based on a single expert applying the workflow to a single clinical topic, and validation across multiple experts, topics, and institutional contexts is needed before generalizability can be established.

## Introduction

Digital health organizations face a strategic paradox when deploying artificial intelligence (AI) for clinical content development. Traditional expert-led methods protect clinical safety, but they can take weeks to months, creating a knowledge translation gap that delays updated guidance [1,2]. AI can synthesize quickly, yet hallucinations can produce plausible but medically inaccurate advice, creating unacceptable liability for leaders accountable for patient safety [3,4]. Managing this speed-safety trade-off has become a core executive challenge. Recent evidence suggests that successful AI transformation requires multidimensional leadership that balances technological opportunities with stakeholder needs [5]. The emergence of multiagent AI systems and autonomous workflows has further intensified this challenge, as organizations must now govern not just individual AI tools but also complex, interacting systems of AI agents [6,7]. This study addresses strategic decision-making under AI-related uncertainty and introduces an actionable governance workflow for health care leaders.

This strategic dilemma is particularly acute in high-stakes clinical scenarios. Consider pediatric febrile seizures—the most common convulsive disorder in childhood, affecting 2%-5% of children between ages 6 months and 5 years [8-10]. Although most febrile seizures are benign and do not cause long-term neurological sequelae [11,12], witnessing one’s child suddenly lose consciousness, stiffen, and tremble is a shocking experience that causes extreme anxiety and fear for parents. In such a high-stress situation, parents need an answer to 1 urgent question: “What do I do right now?”

While professional clinical practice guidelines from organizations such as the American Academy of Pediatrics (AAP) and the National Institute for Health and Care Excellence (NICE) exist [12,13], they are designed for clinicians and do not directly provide actionable guidance for a layperson in a crisis. For instance, a guideline might state, “consider neurodiagnostic evaluation for a child with a complex febrile seizure.” What a parent needs is “clinical guidance”: clear, actionable instructions such as “call 911 immediately if the seizure lasts longer than 15 minutes” or “lay the child on their side.” This gap between expert knowledge and parent-actionable guidance represents a critical challenge in pediatric emergency care.

The traditional process of translating complex medical guidelines into simple, actionable algorithms is resource-intensive, typically requiring multidisciplinary teams working over several weeks to months [14,15]. This knowledge translation bottleneck delays the delivery of potentially life-saving information to families who need it most. As medical evidence evolves, these manually developed materials can quickly become outdated, creating a perpetual cycle of revisions. Recent analyses of clinical practice guideline development timelines have confirmed that the process often takes between 1 and 3 years from conceptualization to publication [16].

Recent advances in large language models (LLMs) have demonstrated significant potential for accelerating medical knowledge synthesis [17]. Their direct application in creating clinical guidance, however, faces a critical challenge: the risk of “hallucination,” where an AI confidently generates plausible but medically inaccurate information [3]. Recent studies document hallucination rates of 1.47% in AI-generated clinical notes, with 44% of these constituting major errors affecting diagnosis or management [18]. While this rate may appear low, in the context of high-stakes pediatric emergencies, where zero tolerance for error is required, even a single such error could have life-threatening consequences. For that reason, deterministic, rule-based algorithms with clear evidence traceability remain essential for ensuring safety [3]. Several recent frameworks have proposed structured approaches to evaluating and mitigating AI-generated clinical content risks, emphasizing the need for systematic human oversight rather than post hoc review [19,20].

For digital health leaders, hallucination risk translates into organizational uncertainty and a critical barrier to adoption. The challenge is strategic: harness AI’s speed without outsourcing accountability for patient safety.

This study describes the development process and formatively evaluates Semiautomatic Clinical Algorithm Development (S-ACAD), an organizational governance framework that positions continuous human oversight as the core management strategy for navigating AI-enabled uncertainty. S-ACAD leverages AI for rapid data collection and synthesis, freeing the expert to focus on high-stakes clinical validation and safety assurance. To explore the feasibility of this approach and make the speed-safety trade-offs explicit, we prospectively applied S-ACAD to pediatric febrile seizures and benchmarked it against a fully automated variant (Fully Autonomous Clinical Algorithm Development [F-ACAD]), providing leaders with preliminary evidence for this strategic choice.

## Methods

### Study Design and Data Sources

**Figure 1:**
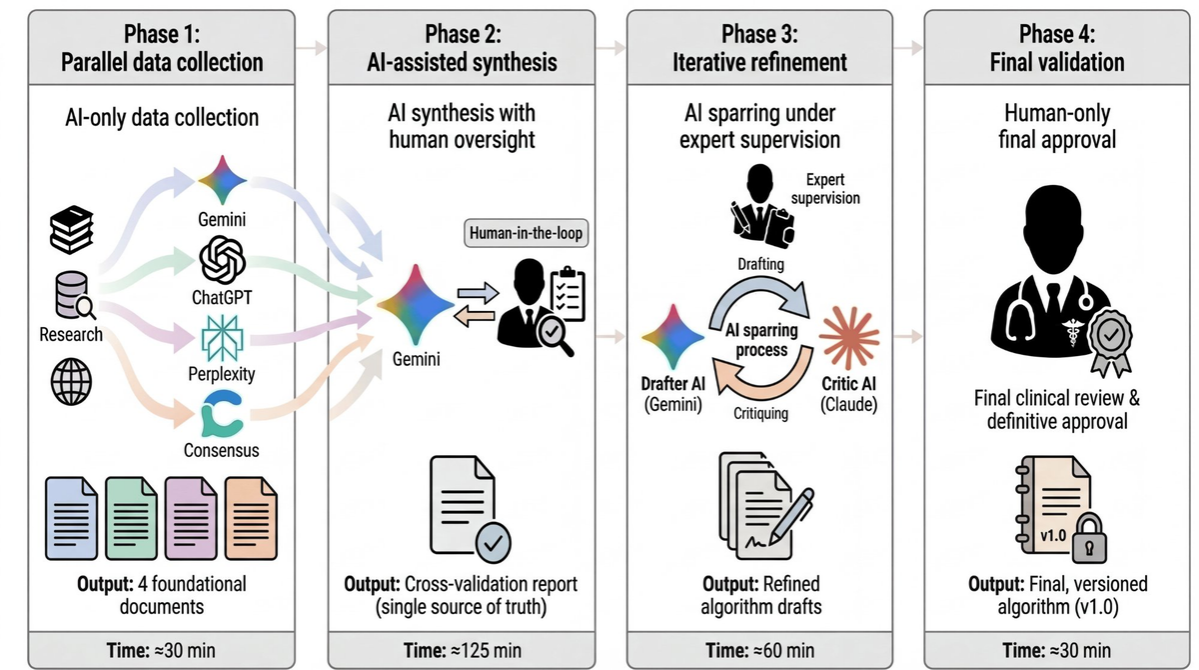
Overview of the Semiautomatic Clinical Algorithm Development framework workflow. AI: artificial intelligence.

This prospective, single-day case study was conducted on July 25, 2025, to execute and document the S-ACAD workflow for developing pediatric febrile seizure guidance (Figure 1). The entire process was designed to be transparent and reproducible, with all artifacts documented in Multimedia Appendices 1-8. This study was informed by the SQUIRE 2.0 (Standards for Quality Improvement Reporting Excellence) reporting guidelines [21], adapted for the formative evaluation of a health care quality improvement workflow (Multimedia Appendix 9).

Data were collected using the “deep research” modes of Gemini 2.5 Pro (Google LLC; accessed July 25, 2025), ChatGPT (model o3; OpenAI; accessed July 25, 2025), Perplexity Pro (Perplexity AI, Inc; accessed July 25, 2025), and Consensus (Consensus; accessed July 25, 2025). The critical review in phase 3 was performed in the “deep thinking” mode of Claude Opus 4 (Anthropic PBC; accessed July 25, 2025). All AI systems were accessed via subscription-based web interfaces with default configurations on the specified date.

### Phase 1: Parallel Foundation Data Collection Using Multiple AIs

To rapidly build a comprehensive and multifaceted information base on pediatric febrile seizures, 4 AI services with different strengths were used in parallel. This strategy was designed to compensate for the potential biases of a single AI model and to secure both the breadth and depth of information simultaneously. To maximize the capabilities of each AI model, customized prompts were designed. The prompts were standardized to include the following elements: a clear role definition (eg, “You are a medical information specialist...”), specification of a concrete output format, key information categories to be included, and a request to specify the level of evidence. Detailed prompts are provided in Multimedia Appendix 1. These 4 services were specifically chosen for their distinct strengths: Gemini and ChatGPT for generating comprehensive foundational text; Perplexity for its focus on citing recent academic literature and guidelines; and Consensus for its ability to extract findings from systematic reviews. Conceptually, this parallel, multimodel querying aligns with the “LLM Council” practice—posing the same prompt to multiple LLMs and comparing their outputs—to surface disagreement early and reduce single-model bias [22].

### Phase 2: Expert-Led, AI-Assisted Cross-Validation

The role of the “human expert” throughout the S-ACAD workflow was performed by the first author (SHA), a board-certified physician with more than 10 years of clinical and medical informatics experience. It is important to acknowledge that SHA is also the originator of the S-ACAD methodology, which introduces the potential for researcher bias. This limitation is further discussed in the “Discussion” and “Conflicts of Interest” sections.

The expert-led cross-validation process began with the human expert conducting a primary review of the 4 AI-generated outputs from phase 1 to check for clinical appropriateness and obvious errors. Subsequently, all 4 documents were fed into an LLM (Gemini), which was instructed to automatically identify points of consensus, conflict, and unique information among the sources and to assign reliability tags, generating a “Cross-Validation Synthesis Report.” This report served as the *single source of truth* in the subsequent phases.

### Phase 3: AI-Human Collaborative Algorithm Generation and Refinement

An initial draft of the algorithm was developed through a cyclical refinement process of “AI generation → AI critique → human revision.” A draft was generated in a decision-tree format by inputting the 5 documents created up to phase 2 into the Gemini model. The resulting draft was then presented to a second AI model (Claude), known for its strengths in critical analysis, to generate a “critical review report” that identified logical flaws, user experience (UX) issues, and missing scenarios (AI sparring). In S-ACAD, we operationalize this idea as a structured drafter-critic pattern (Gemini as drafter; Claude as critic), with the licensed clinician retaining final accountability as the human-in-the-loop. This reframes multimodel comparison as an internal governance control rather than an ad hoc model ensemble. This approach was designed not only to compensate for the potential biases of a single AI model, but also to serve as an internal quality-assurance mechanism that challenges the human expert’s assumptions. The critique was then fed back into Gemini, and during this process, the human expert served as the “human-in-the-loop,” making final clinical judgments, directly revising safety-related content, and adjusting prompts to iteratively refine the algorithm. Claude was selected for the critic role because of its recognized strengths in nuanced reasoning and critical analysis, providing a robust challenge to the initial draft generated by Gemini.

### Phase 4: Final Expert Review

The human expert conducted a final review of the algorithm draft completed in phase 3, verifying the accuracy of all decision pathways, action guidelines, and evidence-level notations, and approved the content as clinically reasonable and safe for parent-facing use, finalizing version 1.0.

### Data Collection and Metrics

By the prospective study design, the following metrics were recorded in real time: time spent on each phase (in minutes), number of AI prompt iterations (AI calls), number and type of human expert interventions, volume of information generated (word count and number of references), and time spent resolving information conflicts. Human expert interventions were logged in real time. Following the experiment, these 19 interventions were retrospectively coded and categorized by the first author (SHA) into 4 predefined categories: (1) clinical judgment (modifying medical logic or content), (2) safety review (adding warnings or conservative pathways), (3) UX optimization (improving clarity or simplicity for the parent user), and (4) prompt adjustment (refining instructions to the AI). The authors acknowledge that retrospective coding by the originator of the methodology, without independent verification (eg, interrater reliability [IRR] assessment), is a limitation and carries a significant risk of interpretation bias. The resulting distribution of intervention types (see panel B in Multimedia Appendix 10) should therefore be interpreted as an exploratory descriptive analysis rather than validated measurements. However, given the complex medical reasoning required to distinguish between categories such as clinical judgment (eg, resolving conflicting evidence) and safety review, expert clinician involvement was deemed essential for accurate categorization.

### Independent Expert Review

Following completion of the algorithm (version 1.0), we commissioned an independent expert review to assess its clinical validity, safety, and completeness. The algorithm was sent for review to 2 board-certified pediatric specialists who were not involved in the study: 1 primary care pediatrician and 1 pediatric emergency medicine physician. These 2 specialties were deliberately chosen to ensure that the algorithm was evaluated from both a primary care perspective (representing routine management and parent education) and an emergency medicine perspective (representing acute, high-stakes situations). A structured survey instrument was used for the review. The instrument included quantitative assessments on a 5-point Likert scale across 4 domains (clinical accuracy, completeness, safety, and usability for parents), as well as qualitative feedback sections for in-depth comments. Reviewers’ identities were known to the author for the purpose of solicitation; however, all ratings and comments were deidentified before analysis and reporting, and no personal identifiers are presented in the manuscript or its multimedia appendices. The primary objective of this review was not statistical generalization but to obtain in-depth qualitative insights and assess whether any critical safety errors requiring mandatory correction were present.

### Comparative Analysis With a Fully Automated Approach

Following S-ACAD completion, an identical task was performed using F-ACAD via Genspark’s multiagent system. It is important to note that F-ACAD represents an experimental baseline configuration constructed on a specific platform to illustrate the risks of ungoverned automation, rather than a definitive representation of all fully automated approaches. The system autonomously deployed 14 specialized AI agents for data collection, validation, algorithm generation, and refinement, without human intervention beyond the initial task specification. The same clinical objective and scope were provided to ensure a fair comparison. Metrics collected included total time, number of agents deployed, sources reviewed, and critical issues identified by the system’s own AI critics.

### AI Model Parameters and Reproducibility

All AI interactions used subscription-based web interfaces with default configurations, as these platforms do not offer user-controllable sampling parameters (eg, temperature and top-p). No random seeds were set, limiting exact output replication. As these interfaces do not provide persistent build identifiers, we report model names and access dates rather than internal version numbers. However, the workflow structure and human intervention patterns remain reproducible. Hardware specifications are not applicable because all computations occurred on cloud-based services. Complete prompts, outputs, and development logs are provided in Multimedia Appendices 1-4 to support methodological transparency. Given the rapid evolution of AI models, the specific model versions used in this July 2025 study may perform differently or be unavailable in the future. Future applications of S-ACAD should document exact model versions and reevaluate model selection accordingly.

### Generative AI Disclosure

In accordance with JMIR’s policy on generative AI use, we disclose that multiple LLMs were used as integral components of the S-ACAD methodology under evaluation. Specifically, Gemini 2.5 Pro was used for cross-validation synthesis and primary algorithm drafting; ChatGPT (model o3) contributed to foundational data collection during phase 1; Perplexity Pro and Consensus supported literature synthesis; and Claude Opus 4 performed critical review (AI sparring).

To ensure scientific rigor and accountability, human oversight was strictly enforced at every stage (Textbox 1).

Complete prompts and AI-generated outputs are provided in Multimedia Appendices 1-4 to support methodological transparency.

**Application of artificial intelligence in this study.**

**1. Research design and experimental protocols**

The study design, that is, the Semiautomatic Clinical Algorithm Development (S-ACAD) framework architecture, and all experimental protocols were conceived and developed entirely by the human authors without artificial intelligence (AI) assistance. The AI tools were used solely as functional agents to execute these human-defined protocols.

**2. Data collection and literature synthesis**

AI agents performed data retrieval and summarization based on strict, predefined prompts designed by the human expert (see Multimedia Appendix 1). All retrieved sources and synthesized findings were verified against the original documents by the human author (phase 2).

**3. Text generation and algorithm drafting**

While AI models generated the initial text drafts and decision tree structures, every decision node was subject to human review. The human expert retained full editorial control, directly revising safety-critical content and finalizing the algorithm (phases 3 and 4).

**4. Manuscript preparation**

Generative AI tools assisted with manuscript drafting and English-language editing. However, all AI-assisted content was reviewed, verified, and finalized by the human author, who takes full responsibility for the final manuscript.

### Ethical Considerations

This study did not involve patients or patient-level data and used only publicly available medical information for algorithm drafting. To ensure methodological rigor and establish content validity, the algorithm underwent cross-verification by 2 external physicians. No sensitive personal data were collected for research purposes, and reviewer feedback was deidentified before analysis and reporting. Given the absence of patient data and the minimal-risk nature of deidentified expert feedback, formal institutional review board review was not sought. Any future deployment of the algorithm would require appropriate institutional review and regulatory oversight to ensure patient safety.

## Results

### Overview

For decision makers, 3 findings stand out: (1) the workflow’s time profile and where effort concentrates, (2) where human oversight remained nonnegotiable, and (3) what happens when automation is left unguided. The detailed workflow metrics are described below.

### Quantitative Results

The S-ACAD framework was completed in approximately 245 minutes (4 hours and 5 minutes) in a single end-to-end execution, during which 8 AI calls and 19 distinct human interventions were recorded. Table 1 summarizes the phase-level resource use. These time values should be interpreted as single-run operational estimates from 1 complete execution by 1 expert, intended to characterize where effort concentrates across phases rather than as generalizable performance benchmarks. The outcome is inherently dependent on the specific expert’s clinical experience and familiarity with AI tools, as well as the performance characteristics of the AI models used at the time of execution. Notably, phase 2 (cross-validation) accounted for the largest share of time (approximately 125 minutes), highlighting that expert validation and reconciliation of multisource AI output—rather than raw drafting—was the dominant workload in this case. This finding underscores the importance and complexity of synthesizing the vast amount of information generated by multiple AIs through a single AI and having a human expert carefully review and validate the results. By contrast, phase 3 (algorithm generation) had the most human interventions (7 instances), indicating intensive collaboration in which the expert refined the logic and enhanced the safety of the AI-generated draft.

In this representative execution of the workflow, each AI model demonstrated distinct strengths and played a complementary role (Table 2). The web-search–based Perplexity cited the most recent literature with 147 references, making a significant contribution to ensuring the timeliness of the evidence. Gemini generated the most words (n=8420), showing strength in systematically structuring information, while ChatGPT provided a comprehensive overview summarizing the key content. This suggests that a strategy of using multiple AIs in parallel may be effective for securing the quantity, quality, and timeliness of information simultaneously.

**Table 1:** Time and resource consumption by the S-ACAD^a^ workflow phase.^b^

Phase	Time (single-run estimate), minutes	Number of artificial intelligence calls, n	Number of human interventions, n
Phase 1: data collection	≈30	4	4
Phase 2: cross-validation	≈125	1	3
Phase 3: algorithm development	≈60	3	7
Phase 4: final review	≈30	0	5
Total	≈245	8	19

^a^S-ACAD: Semiautomatic Clinical Algorithm Development.

^b^Time values are approximate single-run estimates recorded during 1 complete execution of the S-ACAD framework (July 25, 2025). They are reported to describe the relative workflow burden across phases, not to provide generalizable duration benchmarks. A different expert or clinical topic may yield different time profiles.

**Table 2:** Quantitative characteristics of information generated by each artificial intelligence model in phase 1 (data collection).^a^

Artificial intelligence model	Generated word count, n	Number of cited references, n	Primary contribution
Gemini	8420	81	Structured information
ChatGPT	6890	25	Comprehensive overview
Perplexity	4250	147	Latest guidelines
Consensus	7100	50	Academic evidence

^a^The generated word count was calculated based on the body text of each artificial intelligence’s output. The number of cited references was verified and corrected based on the initial development log and the unique sources specified in each artificial intelligence’s output.

### Algorithm Structure and Characteristics

**Figure 2:**
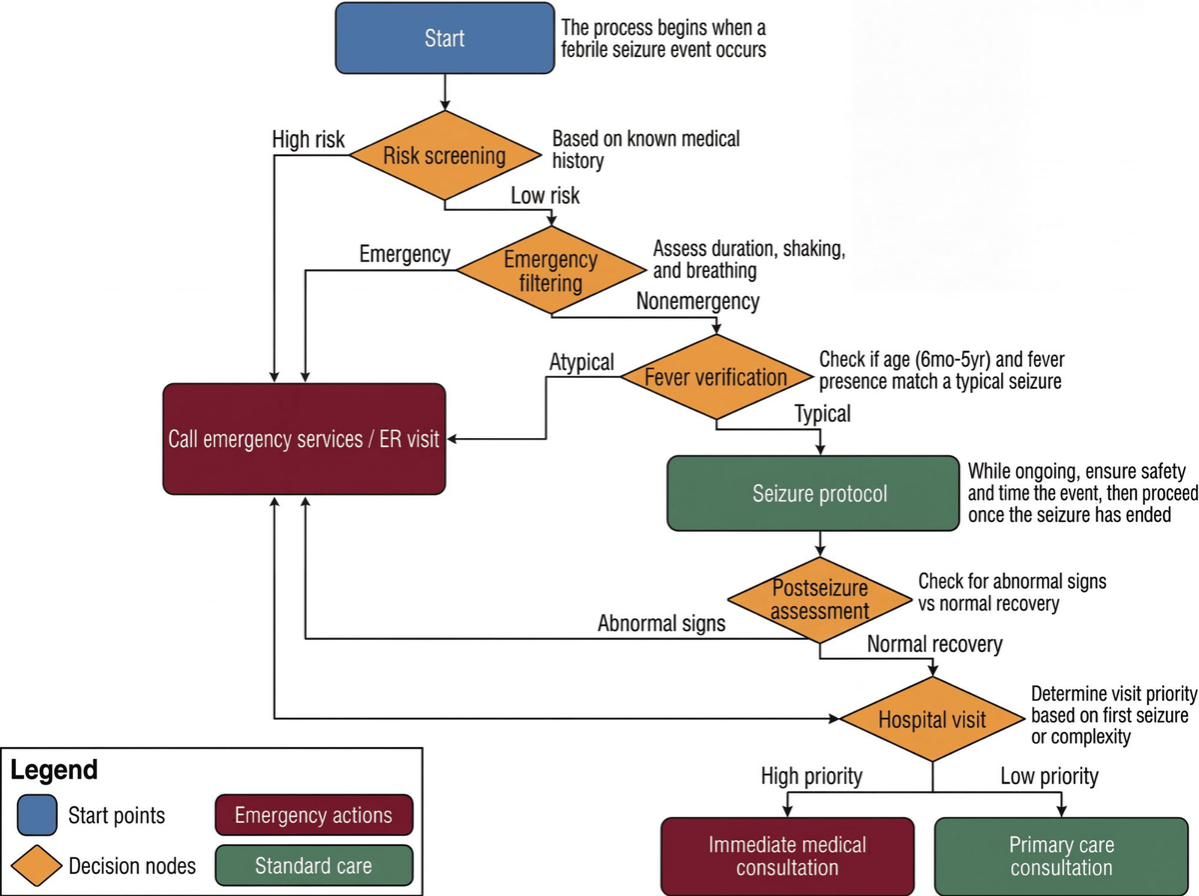
Simplified clinical decision flowchart of the febrile seizure guidance algorithm (version 1.0) developed using the Semiautomatic Clinical Algorithm Development framework. The flowchart guides caregivers through 6 core decision nodes, from initial risk screening to determining the appropriate level of hospital visit. ER: emergency room.

The final developed algorithm is a rule-based decision tree that operates on personalized information and is easy for caregivers to follow. The algorithm’s flow begins at node 0, where the user’s initial information (eg, child’s age, medical history) is entered, and then guides the user through 6 core decision nodes (nodes 1-6; Figure 2). This structure logically progresses from immediately identifying an emergency situation, to managing the seizure as it occurs, to observing after the seizure, and finally to determining the appropriate level of hospital visit required.

The main features of the algorithm are as follows: a total of 6 main decision nodes (excluding initial screening); 23 specific action guidelines; 12 red flag warnings; and pathways optimized to reach a final action within an average of 3-5 steps.

### Evidence Tagging Analysis

To ensure the reliability of the algorithm, all 23 action guidelines were tagged with their level of evidence. The analysis confirmed that 18 (78%) guidelines were based on a “high consensus level (A),” which is consistently recommended across multiple guidelines (Table 3). This indicates that the core content of the algorithm is based on robust medical consensus. There were 4 guidelines (17%) with a “medium consensus level (B)” and 1 (4%) with a “conservative recommendation (C),” which represented a case where the expert made a direct judgment to recommend the safest course of action when evidence was insufficient or conflicting. This transparent evidence labeling not only increases the algorithm’s reliability but also helps clearly identify areas for modification when evidence is updated in the future.

**Table 3:** Distribution of evidence levels within the algorithm.

Level of evidence	Values, n (%)
A (high consensus)	18 (78)
B (medium consensus)	4 (17)
C (conservative recommendation)	1 (4)

### AI Sparring Effectiveness Analysis

The AI sparring phase, a core component of phase 3, appeared to contribute to improving the draft’s quality in this single-case execution. The Claude model, tasked with critical review, identified a total of 16 improvement suggestions for the draft generated by Gemini. Of these, 14 (88%) were adopted into the final algorithm after expert review. The specific improvement suggestions included the following: logic gaps (3, all adopted); UX improvement suggestions (7, 5 adopted); missing scenarios (4, all adopted); and safety enhancements (2, all adopted). These results suggest that AI sparring may serve as a valuable internal quality-assurance mechanism, extending beyond simple error detection to support the algorithm’s completeness, user-friendliness, and safety.

### Analysis of Human Interventions

The descriptive analysis of the 19 human interventions, as categorized retrospectively by the first author (SHA), indicated that clinical judgment (8/19, 42%) and safety review (5/19, 26%) were the most frequent types of input (see panel B in Multimedia Appendix 10). UX optimization accounted for 3 out of 19 (16%) and prompt adjustment for 2 out of 19 (11%). Although these findings are specific to a single case study and are subject to potential interpretation bias due to the lack of independent coding verification (see the “Limitations” section), they highlight the areas in which expert oversight appeared to concentrate during the workflow. Future multiexpert studies with IRR assessment will be necessary to validate these exploratory categorizations.

### Expert Review Results

#### Independent Specialist Validation

The final algorithm underwent a formal review by 2 independent, board-certified pediatric specialists—a pediatric emergency physician (reviewer A) and a primary care pediatrician (reviewer B)—both with 15 years of clinical experience.

#### Safety Review

Crucially, neither reviewer independently identified medically inaccurate sections or critical safety errors requiring mandatory correction. This review served as a vital safety check against potential oversights by the single developer.

#### Qualitative Feedback

The qualitative feedback strongly supported the algorithm’s utility (Multimedia Appendix 7). Reviewer A (emergency physician) stated that the algorithm’s greatest strength was that “it provides comprehensive and accurate guidance...especially for situations requiring attention.” Reviewer B (primary care pediatrician) noted that it “allows caregivers to respond safely at home...which can provide reassurance.” Both rated the algorithm as “much better” than existing educational materials and recommended it as “usable after minor revisions” for a parent-facing service, pending successful usability testing.

#### Quantitative Overview

Although not intended for statistical generalization, given the small sample size (N=2), quantitative assessments were also conducted (Multimedia Appendix 6). Both reviewers strongly agreed (providing the highest possible rating) on all items within the clinical accuracy and safety domains. Although ratings for completeness and usability were generally high, reviewer A noted a concern regarding the “practical executability” of certain guidelines under stress (scoring 3/5 on item 4-2). Overall, the reviewers assigned high ratings for the algorithm’s clinical validity (9.0/10 and 9.5/10 by reviewers A and B, respectively). Full raw data are available in Multimedia Appendix 8.

### Comparative Analysis: S-ACAD Versus F-ACAD

The F-ACAD system, powered by the Genspark Super Agent, completed the entire algorithm development task in 67 minutes and 58 seconds—approximately 3.6 times faster than S-ACAD’s 245 minutes. However, this substantial reduction in time was accompanied by notable quality trade-offs. Table 4 summarizes the key differences between the 2 approaches.

Although F-ACAD reviewed more sources and produced a more complex algorithm, its own AI critics identified 17 issues requiring correction, including 9 high-priority concerns related to US standard-of-care compliance, emergency-response clarity, and parent-friendly communication. These were precisely the areas in which S-ACAD’s 19 human interventions appeared most relevant.

To directly compare the shortcomings of the fully automated approach with the contributions of the human expert, the 17 issues identified by F-ACAD’s critics were mapped to the categories of the 19 human interventions performed in S-ACAD. The analysis suggests a correspondence between F-ACAD’s weaknesses and the areas in which human clinical judgment and safety oversight were most needed (Table 5).

**Table 4:** Comparison of S-ACAD^a^ and F-ACAD^b^ approaches.

Characteristic	S-ACAD	F-ACAD
Total time (minutes)	≈245	≈68
Human interventions, n	19	1 (initial task specification only)
AI^c^ calls/agents, n	8 AI calls	14 autonomous agents
Sources reviewed, n	18	25
Algorithm nodes, n	22	25
Critical issues identified, n	N/A^d^ (addressed by human in-process)	17 (9 high and 8 medium)

^a^S-ACAD: Semiautomatic Clinical Algorithm Development.

^b^F-ACAD: Fully Autonomous Clinical Algorithm Development.

^c^AI: artificial intelligence.

^d^N/A: not applicable.

**Table 5:** Mapping of high-priority issues in F-ACAD^a^ to human intervention types in S-ACAD^b^.

F-ACAD high-priority issue category	Example issue identified by F-ACAD critics	Corresponding human intervention type in S-ACAD
Clinical accuracy	Inadequate differentiation between emergency room and primary care physician visits	Clinical judgment (8/19, 42%)
Safety and emergency response	Inconsistent emergency response timing (3-5-minute ambiguity)	Safety review (5/19, 26%)
Parent-centric communication	Complex medical terminology without lay explanations	User experience optimization (3/19, 16%)
Logical completeness	Missing age-specific nuances for children <12 months	Clinical judgment (8/19, 42%)

^a^F-ACAD: Fully Autonomous Clinical Algorithm Development.

^b^S-ACAD: Semiautomatic Clinical Algorithm Development.

## Discussion

### Summary of Findings

This proof-of-concept study set out to describe and formatively evaluate the S-ACAD framework as a governance approach for AI-assisted clinical algorithm development. The primary objectives were to assess the feasibility of the “Human-in-the-Loop” model and to explore the speed-safety trade-off relative to full automation. In this single-case execution, the S-ACAD framework produced a parent-actionable febrile seizure algorithm in approximately 245 minutes. Two independent pediatric specialists did not identify critical safety errors requiring mandatory correction, and both recommended the algorithm as usable with minor revisions. The fully automated comparator (F-ACAD), although approximately 3.6 times faster, generated 17 issues (9 high priority) that overlapped with the categories in which human oversight was concentrated in S-ACAD. These findings, although preliminary and specific to this single case, suggest that continuous expert governance may help mitigate the safety risks associated with fully automated approaches.

### Interpretation and Implications

#### Active Governance as a Management Strategy for Navigating AI-Enabled Uncertainty

A central challenge for digital health leadership is managing AI-enabled uncertainty. S-ACAD addresses this challenge by redefining the human-in-the-loop. Rather than serving merely as a passive final reviewer, the human expert engages in “active governance”—directing the AI, applying critical clinical judgment, and enforcing ethical oversight and safety throughout the development lifecycle. In this study, the 19 documented interventions (see the “Analysis of Human Interventions” section and Multimedia Appendix 10) illustrate what this governance looked like in practice and appear to map to the safety and nuance failures observed under full automation (F-ACAD).

The exploratory analysis of the 19 human interventions, as categorized by the first author, indicated that clinical judgment (8/19, 42%) and safety review (5/19, 26%) constituted the majority of interventions (see panel B in Multimedia Appendix 10). Although this categorization requires independent validation, it suggests that AI may currently lack the nuanced understanding of clinical context, ethical considerations, and patient safety required for reliable guidance [17]. S-ACAD leverages this dynamic, elevating the expert from a data collector to an augmented “clinical strategist” and “safety guarantor.”

A critical example illustrates the potential importance of this governance. In phase 3, the AI initially generated a protocol allowing parents to directly administer prescribed emergency medication (eg, diazepam) if a seizure lasted more than 5 minutes. The human expert intervened to modify this pathway, mandating that parents call 911 for real-time instructions before administration (Multimedia Appendix 4). This intervention, driven by considerations of liability, situational stress, and the need for professional guidance during medication use, exemplifies a safety-critical judgment that the automated system failed to make.

Other key interventions included providing nuanced guidance on antipyretics based on conflicting evidence [23,24] and adding specific pathways for atypical presentations (eg, first seizure over age 5). These examples suggest that human-in-the-loop may be essential for mitigating AI limitations, managing uncertainty, and ensuring that the final product is clinically sound and safe. The quantitative impact of such oversight is supported by recent literature: a controlled study reported that diagnostic accuracy among the best-performing clinicians decreased when AI advice was introduced (from 87.3% to 77.1%), highlighting how AI-related “false conflict” can negatively affect expert performance and underscoring the need for structured human oversight [25]. This provides external context for the potential value of S-ACAD’s human interventions.

#### Efficiency Considerations

In this proof-of-concept case, S-ACAD produced a clinically reviewed draft in just over 4 hours. Relative to published descriptions of traditional manual synthesis timelines—often reported in weeks and involving 60-200 person-hours of multidisciplinary team effort [14,15]—this suggests the potential for a reduction in turnaround time, although the magnitude remains illustrative in the absence of a concurrent traditional control. As we did not conduct a parallel traditional control process on the same topic, the magnitude of any efficiency gain should be interpreted as illustrative rather than definitive. The 245-minute figure reflects a single execution by 1 expert, who was also the methodology’s originator and therefore had extensive familiarity with the workflow; a different expert or a different clinical topic might require more or less time.

A potential strategic advantage of S-ACAD is its capacity to support “perpetual updates” of clinical guidance. In the current evidence ecosystem, there is often a substantial lag between the publication of new evidence and its incorporation into patient-facing materials—the knowledge translation gap [14]. If the efficiency observed in this case generalizes to other contexts, S-ACAD’s accelerated timeline could help reduce this gap, keeping guidance aligned with the latest evidence and strengthening patient safety. This capability also aligns with learning health systems, in which the continuous integration of new evidence into clinical practice is a core operational principle [26].

The efficiency gains appear to arise from restructuring the development workflow. Traditional methods are often slowed by sequential reviews and delayed committee consensus. S-ACAD uses parallel AI processing and “AI sparring” (phase 3) to convert a linear peer-review cycle into rapid iteration, potentially reducing waiting times associated with sequential review cycles.

Notably, the AI-assisted synthesis phase took the longest (125 minutes), reflecting the intensive work required for expert validation of AI-generated sources. This finding underscores a critical distinction: S-ACAD may improve efficiency not by removing the human expert, but by reallocating effort away from laborious data collection and toward high-value validation and synthesis. This “cognitive reallocation” may represent the methodology’s core strategic advantage. For digital health management, it suggests a potential talent realignment in which expert roles shift from routine data collection to high-stakes validation and governance. Although this expert validation remains the rate-limiting step, it is also the methodology’s most critical safety feature.

#### Generalizability of the Methodology

The core elements of the S-ACAD framework show potential applicability, particularly in clinical areas in which clear guidelines exist [13,27], logical expression in a decision tree is feasible, and the level of evidence is well established. This makes the methodology potentially suitable for developing parent-actionable guidance for acute pediatric emergencies commonly encountered at home, extending well beyond febrile seizures.

For example, other acute pediatric conditions with well-defined clinical guidelines and clear decision logic—such as anaphylaxis management or minor head injury triage—may be amenable to this workflow, although this remains to be empirically tested.

By contrast, there are areas in which the application of this methodology may face significant challenges. For topics involving rapidly advancing treatments or emerging technologies, there may be a lack of consensus evidence, meaning that the data available for AI synthesis are themselves limited. Furthermore, in areas such as mental health, where cultural context and the therapeutic relationship are central, or in chronic disease management, where long-term lifestyle and personal preferences have substantial influence, it is difficult to create comprehensive guidance based solely on text-based evidence. In these contexts, S-ACAD should be used as an initial data-collection and structuring tool, but expert qualitative judgment and user-participation processes must be further strengthened. Consistently, agent benchmarks (eg, SWE-Bench Pro, GAIA) demonstrate sharp performance degradation as tasks require multistep reasoning, tool use, and cross-source synthesis—conditions intrinsic to clinical algorithm development [28,29].

#### Strategic Choice: Comparing Governance Models Across the Automation Spectrum

Strategic decision-making under AI-related uncertainty requires digital health leaders to select a governance model that balances efficiency and rigor. Our comparative analysis of 3 approaches—traditional (human-only), F-ACAD (AI-only), and S-ACAD (hybrid)—provides a preliminary decision framework to inform this management choice.

Traditional methods (human-only) prioritize rigor, involving extensive literature review and expert consensus. However, this approach is characterized by low efficiency, often taking weeks or months [13,14], which can lead to outdated guidance and delayed knowledge translation.

The fully automated approach (F-ACAD, AI-only) was fast, completing the task in approximately 68 minutes. However, that speed was accompanied by a clear governance gap. Although F-ACAD reviewed a larger volume of sources (25 vs 18 in S-ACAD), this recall-oriented capacity did not translate into clinical precision. The system generated 17 critical issues, including 9 high-priority concerns related to clinical safety, nuance, and communication (Table 5). Many of these issues overlapped with the areas in which human intervention was most frequent in S-ACAD, providing some triangulation for the identified intervention categories despite the limitation of single-researcher coding. This pattern is consistent with broader benchmarks suggesting that fully autonomous agents continue to struggle with end-to-end, real-world tasks; the Remote Labor Index [30] reported an autonomous completion rate of only 2.5% across 240 freelance tasks. Taken together, the 17 critical issues appear less like an isolated failure and more like a predictable consequence of generating patient-facing guidance without continuous human governance.

S-ACAD represents a deliberate strategic choice: a hybrid governance model that integrates AI for speed while embedding human expertise to manage uncertainty and ensure safety. For digital health leaders, this comparison clarifies the stakes: fully automated approaches (F-ACAD) offer speed but may introduce unacceptable clinical risk, whereas traditional approaches offer safety but may not keep pace with rapidly evolving evidence. These preliminary findings suggest that a redesigned workflow, in which human expertise is strategically allocated to governance and validation, may achieve both the efficiency required for the digital age and the rigor essential for patient care.

#### Strategic Implications for Digital Health Management

##### Organizational Implementation Strategy

Adoption of S-ACAD requires strategic changes in workforce structure and resource allocation. Organizations implementing this framework should consider the following:

- Talent realignment: Shifting expert roles from routine data collection to high-stakes validation and governance.
- Infrastructure requirements: Ensuring access to multiple AI platforms, version-control systems, and transparent documentation workflows.
- Training and change management: Preparing clinical experts to direct AI rather than compete with it.
- Pilot implementation: Beginning with low-risk clinical topics before scaling to high-stakes applications.

##### Preliminary Resource and Implementation Considerations

**Figure 3:**
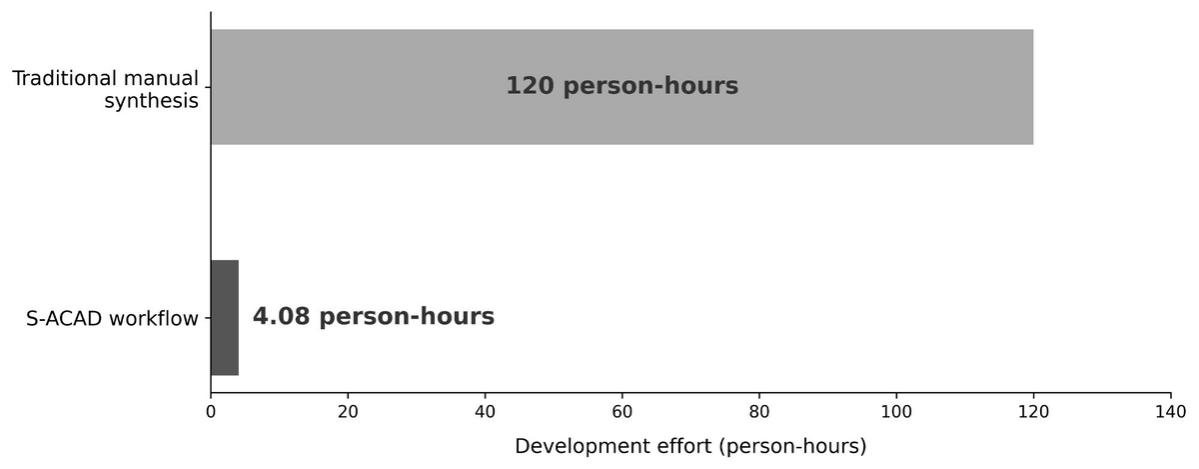
Comparison of development effort between traditional manual synthesis (120 person-hours) and the Semiautomatic Clinical Algorithm Development (S-ACAD) workflow (4.08 person-hours). Traditional estimates are based on published literature [14,15]. S-ACAD time was empirically measured during a single execution. S-ACAD: Semiautomatic Clinical Algorithm Development.

Although this single-case study cannot provide a formal economic evaluation, the observed time profile offers an illustrative resource comparison (Figure 3). Traditional guideline translation typically requires multidisciplinary teams (2-5 clinical experts, medical writers, and editors) working 60-200 person-hours over several weeks [14,15], whereas S-ACAD in this case achieved same-day completion by a single expert in approximately 4 hours, alongside subscription access to multiple AI platforms. However, organizational resource implications would also depend on initial setup costs, AI subscription expenses, quality-assurance overhead, and long-term maintenance. Multisite implementation studies with prospective time tracking and cost accounting are needed to quantify the organizational value proposition across diverse institutional contexts.

##### Decision Framework for Leaders

Our case suggests 3 potentially “nondelegable” checkpoints: (1) evidence traceability before synthesis becomes a single source of truth; (2) safety-first review during decision-node generation; and (3) independent clinical review before deployment. In practice, leaders can separate “speed layers” (parallel collection and drafting) from “accountability layers” (validation, safety, and external review) to capture efficiency without transferring clinical risk to automation.

##### Anticipated Implementation Barriers

Despite its potential advantages, organizational adoption of S-ACAD may face several practical barriers:

- Clinical expert resistance: A traditional culture of slow, deliberative peer review may perceive S-ACAD’s speed as “cutting corners.”
- IT and infrastructure gaps: Adoption requires organizational access to multiple AI platforms and version-control systems.
- Regulatory and liability uncertainty: Although S-ACAD’s human governance and transparent documentation are designed to mitigate risk, legal frameworks for AI-assisted content generation are still evolving.
- ROI measurement challenges: The primary benefits, such as closing the knowledge translation gap, are often intangible and more difficult to quantify than direct cost savings.

#### Beyond Accuracy: Practical Executability, Ethical Considerations, and the Need for User-Centered Design

The strategic implications of this work extend beyond development efficiency to implementation readiness. Although S-ACAD may produce clinically accurate guidance, accuracy alone is insufficient for real-world effectiveness. This mirrors the implementation gap widely reported in health care AI: many initiatives demonstrate high laboratory accuracy yet fail to scale beyond pilot settings in practice, often because of challenges related to usability, governance, and workflow integration [28]. By embedding governance considerations and workflow compatibility during development rather than addressing them post hoc, the S-ACAD framework represents a proposed strategy to mitigate these systemic risks.

A critical insight from the expert review highlights this challenge. Reviewer A questioned the feasibility of the “wait for 5 minutes” rule, noting that caregivers, under extreme stress, might be unable or unwilling to wait, regardless of the guideline (Multimedia Appendix 7). This observation underscores a gap that S-ACAD, as an evidence-synthesis methodology, cannot bridge on its own.

These findings confirm that S-ACAD is intended as a development tool, not a deployment solution. Even a clinically validated algorithm requires a rigorous subsequent phase of user-centered design—including usability testing with diverse caregivers, cultural adaptation, and attention to health literacy and equity considerations—before it can be responsibly deployed.

### Limitations

#### Overview

This study, as a proof-of-concept, has several important limitations that should be considered when interpreting the results.

#### Single-Case Study Design

This is an “n-of-1” study: a single expert applied the workflow to a single clinical topic. The time profile reported here (approximately 245 minutes) reflects a single-run operational measurement from 1 complete execution by 1 expert and cannot characterize variability across topics, teams, or organizational settings. It is unclear whether this time represents a best-, average-, or worst-case scenario. The 245-minute figure may have been influenced by the expert’s familiarity with the methodology (as its originator) and by the clinical topic’s suitability for decision-tree structuring. Different experts—particularly those less familiar with AI tools or the S-ACAD workflow—might require substantially more time. Similarly, clinical topics with less clearly defined guidelines or more complex decision logic might yield different results. We therefore present the timing primarily to describe the relative distribution of effort across phases rather than to claim generalizable efficiency benchmarks. A more rigorous approach would involve multiple experts applying the workflow to multiple clinical topics to establish variability and generalizability.

#### Researcher Bias

A substantial potential for researcher bias exists, encompassing both execution bias (during development) and interpretation bias (during analysis). The first author (SHA) is both the originator of the S-ACAD methodology and the human expert who executed the workflow, which inevitably influenced workflow execution. The retrospective coding of the 19 human interventions (see panel B in Multimedia Appendix 10) was performed solely by SHA, without independent verification through IRR assessment. The resulting distribution of intervention types (eg, clinical judgment, 8/19, 42%, or safety review, 5/19, 26%) therefore reflects the first author’s interpretation and may be influenced by confirmation bias. These proportions should be interpreted as exploratory rather than as objective measurements. This single-expert dependency also means that the final algorithm reflects both the S-ACAD framework and the expert’s individual clinical judgments; a different expert might intervene differently, leading to different outputs and development timelines.

To partially mitigate these bias risks, 2 design features were intentionally embedded (Textbox 2).

**Design features embedded.**

**1. Artificial intelligence sparring as a bias-challenge mechanism**

Phase 3’s artificial intelligence (AI) sparring was specifically designed to provide algorithmic critique independent of the developer’s assumptions. The high adoption rate (14/16, 88%) indicates that AI-generated critiques were frequently incorporated after expert review, which may reflect the mechanism’s capacity to challenge and refine initial decisions.

**2. Independent blinded validation**

The external expert review by 2 board-certified pediatricians, conducted blind to the development process, served as a safety check. Neither reviewer identified critical safety errors requiring mandatory correction, providing some reassurance that execution bias did not compromise clinical safety in this single-case run. Although these mechanisms cannot eliminate bias entirely, they represent a deliberate methodological strategy to bound its impact. Future multiexpert implementations should include interrater reliability data to more fully characterize the role of individual expert variation.

#### Lack of Concurrent Traditional Comparison

The comparison with traditional methods is based on literature estimates (60-200 person-hours) rather than on a concurrently conducted traditional process [14,15]. Without a direct comparison using the same topic and comparable expertise, the efficiency implications remain illustrative rather than empirically validated. No specific percentage efficiency gain should be inferred from this proof-of-concept. Similarly, the comparison with F-ACAD highlights potential risks associated with inadequate governance but does not establish the optimal level or method of human oversight.

#### Reproducibility Challenges

All AI interactions were conducted using subscription-based web interfaces with default configurations, as these platforms do not provide user-controllable sampling parameters. No random seeds were set, limiting exact output replication. The rapid evolution of AI models presents an additional challenge; the specific model versions used in this July 2025 study may perform differently or may be unavailable in the future. Future applications of S-ACAD should document the exact model versions used and reevaluate model selection as platforms evolve.

#### External Validation Limitations

The external review was conducted by only 2 reviewers, which lacks sufficient statistical power for generalization. The resulting scores should therefore be interpreted as qualitative indicators within this proof-of-concept study rather than as statistically validated metrics.

#### Contextual Limitations

The algorithm developed in this study reflects US-centric emergency response protocols (eg, “911” as the emergency number and specific medication availability). Health care systems vary substantially across countries in emergency services, medication regulations, and clinical practice norms. Organizations implementing S-ACAD in different contexts should incorporate an explicit localization phase.

#### Scope of Conclusions

Finally, although this proof-of-concept suggests the feasibility and safety of S-ACAD as a governance framework for algorithm development, it does not constitute validation of its effectiveness as an organizational management strategy. The strategic implications discussed—including talent realignment, resource considerations, and organizational adoption—are derived from this single case and require empirical testing through multiinstitutional implementation studies. Decision makers considering the adoption of S-ACAD should view this work as providing a structured framework and preliminary evidence, rather than definitive proof of organizational benefit.

### Future Research Directions and Broader Implications

To address these limitations and advance the field of AI-assisted clinical content governance, several key directions emerge:

First, *empirical validation with end users* is the most critical next step. Simulation-based usability testing with actual caregivers is essential to evaluate the algorithm’s safety and effectiveness for its target audience and to address sociocultural barriers to adherence.

Second, *multicenter, multiexpert, multitopic validation* is needed. S-ACAD should be applied repeatedly to diverse clinical topics by multiple experts across different institutions. By systematically collecting development time data (using predefined time-tracking procedures) and comparing results against traditional methods as a control, a more robust statistical foundation for the methodology’s efficiency can be established, including CIs.

Third, *rigorous comparative studies and exploration of the automation spectrum* are warranted. Different hybrid human-AI configurations—including hypothesized intermediate models between S-ACAD and F-ACAD—should be systematically evaluated to define the optimal balance between automation and expert oversight.

Fourth, *implementation science and organizational studies* are required to validate S-ACAD as a management strategy. This includes multiinstitutional implementation studies assessing adoption barriers and stakeholder acceptance, cost analyses and economic evaluations from a health system perspective, and longitudinal studies evaluating sustainability and scalability.

More broadly, the findings of this study contribute to a growing body of evidence that governance of AI in health care cannot be reduced to a binary choice between human-only and AI-only approaches. Instead, effective governance requires deliberate, continuous alignment of human expertise with AI capabilities—what we have termed “active governance.” As AI systems become increasingly capable, the role of the human expert is not diminished but elevated to a higher-order function: ensuring that AI-generated outputs meet the safety, ethical, and contextual standards that patients and families deserve. The S-ACAD framework, although requiring further validation, offers an operationalizable model for this governance approach that health care organizations can adapt and test within their own institutional contexts.

### Conclusions

In this single prospective case study, the S-ACAD framework produced a parent-actionable febrile seizure algorithm in just over 4 hours while preserving evidence traceability and incorporating independent clinical review. Two independent pediatric specialists did not identify critical safety errors requiring mandatory correction, suggesting that continuous expert governance during AI-assisted development may help maintain clinical safety standards. Although limited to a single expert and clinical topic, these preliminary findings suggest a practical governance pathway for digital health teams that require speed but cannot outsource accountability.

The key insight from this formative evaluation is not the time metric itself, but the redistribution of expert effort: S-ACAD assigns AI to rapid collection and drafting while reserving expert effort for validation, governance, and safety—areas in which human judgment appeared most consequential. Critical next steps include multiinstitutional implementation studies to evaluate S-ACAD as an organizational strategy across diverse health care settings, clinical domains, and institutional contexts. More broadly, the “active governance” paradigm demonstrated here—embedding continuous human oversight within AI-accelerated workflows rather than relying on post hoc review—may offer a generalizable model as health care organizations navigate emerging regulatory frameworks for AI-assisted clinical tools. As both the EU AI Act and Food and Drug Administration (FDA) guidance on AI/machine learning–based Software as a Medical Device increasingly emphasize human oversight and algorithmic transparency, governance approaches such as S-ACAD that operationalize these principles during development, rather than retrofitting them after deployment, may become essential components of responsible digital health innovation.

## Appendix

Multimedia Appendix 1 Detailed prompts used for each artificial intelligence model.

Multimedia Appendix 2 Full text of the cross-validation synthesis report.

Multimedia Appendix 3 Complete flowchart of the final algorithm (version 1.0).

Multimedia Appendix 4 Detailed log of the artificial intelligence sparring and iterative development process.

Multimedia Appendix 5 Comparative analysis of the Fully Autonomous Clinical Algorithm Development methodology and results.

Multimedia Appendix 6 The expert evaluation form.

Multimedia Appendix 7 Anonymized qualitative feedback from external experts.

Multimedia Appendix 8 External review raw data (quantitative scores).

Multimedia Appendix 9 The SQUIRE (Standards for Quality Improvement Reporting Excellence) 2.0 reporting checklist.

Multimedia Appendix 10 Artificial intelligence sparring effectiveness and human intervention analysis. Panel A presents the number of issues identified by the artificial intelligence critic versus the number adopted after expert review, by category. Panel B shows the distribution of all 19 human expert interventions recorded across the Semiautomatic Clinical Algorithm Development workflow.

## Abbreviations

AAP: American Academy of Pediatrics
AI: artificial intelligence
F-ACAD: Fully Autonomous Clinical Algorithm Development
FDA: Food and Drug Administration
GAIDeT: Generative AI Delegation Taxonomy
IRR: interrater reliability
LLM: large language model
NICE: National Institute for Health and Care Excellence
S-ACAD: Semiautomatic Clinical Algorithm Development
SQUIRE: Standards for Quality Improvement Reporting Excellence
UX: user experience

## Data Availability

All data generated and analyzed during this study are included in this manuscript and its multimedia appendices. The detailed prompts used for each artificial intelligence (AI) model (Multimedia Appendix 1), the full cross-validation synthesis report (Multimedia Appendix 2), the complete algorithm flowchart (Multimedia Appendix 3), the detailed AI sparring and development process log (Multimedia Appendix 4), the Fully Autonomous Clinical Algorithm Development (F-ACAD) comparative analysis data (Multimedia Appendix 5), the expert evaluation form (Multimedia Appendix 6), anonymized qualitative feedback (Multimedia Appendix 7), and quantitative review scores (Multimedia Appendix 8) are provided as multimedia appendices to ensure transparency and support reproducibility of the described workflow.
