# Supplementary material for: Strategic Governance of Artificial Intelligence–Enabled Clinical Algorithm Development: Formative Evaluation of the Semiautomatic Clinical Algorithm Development Framework
Source: JMIR Form Res. 2026 Mar 12;10:e90273. doi: 10.2196/90273 (PMC13022556; doi:10.2196/90273)
Supplement: Multimedia Appendix 1 [file formative_v10i1e90273_app1.docx]

This appendix provides the detailed prompts used in Phase 1 (Parallel Data Collection) of the S-ACAD workflow, in both English and Korean, to maximize the capabilities of each AI model and ensure consistency in the outputs.

**1. Prompts for Foundational LLMs (Gemini, ChatGPT)**

**Objective:** To generate a comprehensive, encyclopedic 'Knowledge Base' on pediatric febrile seizures, serving as a baseline document for cross-validation. The focus is on factual description rather than rule-based instructions.

"You are a 'Medical Knowledge Researcher' tasked with building a medical knowledge database based on pediatric neurology textbooks and the latest clinical papers. Please create a comprehensive baseline document on 'Pediatric Febrile Seizures.' This document will be used as a 'Baseline Document' for cross-validation with other information and for expert review. The output should be in an encyclopedic, descriptive format detailing objective facts, NOT in an 'IF-THEN' rule-based format.

I. General Overview

1. Definition: Clinical definition of a febrile seizure.
2. Epidemiology: Peak age, incidence, prevalence.
3. Pathophysiology: Currently known mechanisms of occurrence.
4. Classification: Detailed criteria for simple vs. complex febrile seizures and the clinical significance of each item.

II. Clinical Aspects and Management

1. Risk Factors: Detailed description of factors influencing first occurrence and recurrence (e.g., family history, age, underlying conditions).
2. Management [Before Seizure]:
   - Importance of differentiating the cause of fever.
   - Current medical stance on the use of antipyretics.
3. Management [During Seizure]:
   - Step-by-step first-aid procedures at home.
   - Absolute and relative criteria for calling emergency medical services (e.g., 911).
4. Management [After Seizure]:
   - Characteristics of the post-ictal state and key observation points.
   - Typical questions asked during a medical visit, and types/purposes of possible examinations.

III. Prognosis and Additional Considerations

1. Long-term Prognosis: Rate of transition to epilepsy, impact on neurodevelopment and cognitive function.
2. Recurrence: Recurrence rates and associated factors.
3. Differential Diagnosis: Other conditions that must be distinguished from febrile seizures (e.g., meningitis, afebrile seizures).

IV. [Additional/Enhanced Section] Special Situations and Various Scenarios

1. Considerations by Population:
   - Age: Specific characteristics and precautions for different age groups, such as infants (6-12 months) and children over 5 years.
   - Underlying Conditions: Differences in management for children with developmental delays, epilepsy, heart disease, etc.
2. Considerations by Situation:
   - Location: Management guidelines for non-home environments like daycare centers or during travel.
   - Time: Response strategies when medical access is limited, such as at night or on weekends.
3. Considerations for Different Caregivers:
   - Key information to be conveyed to non-primary caregivers like grandparents or babysitters.

Requirements:

- For each fact, please provide evidence where possible (e.g., 'According to the American Academy of Pediatrics guidelines...').
- Please specify areas of controversy or uncertainty where expert opinions differ or more research is needed as 'Controversial Area' or 'Uncertainty'."

**2. Prompt for Perplexity**

**Objective:** To gather the latest clinical guidelines, systematic reviews, and key clinical summaries with a strong emphasis on citing credible sources.

"I am developing a digital health algorithm for parents/caregivers about pediatric febrile seizures. Please find and summarize comprehensive information with sources on the following topics.

1. Latest Clinical Guidelines (Focusing on 2020-2025)

- American Academy of Pediatrics (AAP) guidelines
- UK National Institute for Health and Care Excellence (NICE) guidelines
- Korean Pediatric Neurological Society guidelines
- Comparison of recommendations from major national pediatric societies (focusing on differences)

1. Results from Systematic Reviews and Meta-Analyses

- Epidemiology, risk factors, recurrence rates
- Management and treatment strategies (e.g., use of antipyretics, anticonvulsants)
- Long-term prognosis (neurodevelopment, risk of transitioning to epilepsy)

1. Summary of Key Clinical Information

- Clear diagnostic criteria for simple vs. complex febrile seizures
- Red flag symptoms requiring immediate medical attention
- Evidence-based first-aid methods that can be done at home

1. Parent Education Materials

- Parent guidance materials distributed by major medical institutions (e.g., university hospitals)
- Common misconceptions and medical facts about febrile seizures

Requirements:

- Please cite the source (institution name, year of publication) for all information.
- If there are conflicting contents between guidelines, please highlight them specifically."

**3. Prompt for Consensus**

**Objective:** To obtain a high-level summary of the scientific consensus on the topic, explicitly highlighting areas of debate or gaps in evidence.

"Please provide a comprehensive literature review covering all of the following items on pediatric febrile seizures.

- Core concepts and definitions
- Key clinical features
- Typical case presentations
- Evidence-based medical responses and management methods
- Common misconceptions
- Summarize the consensus among researchers, and also highlight any areas of controversy or gaps in the evidence."
