# Supplementary material for: Strategic Governance of Artificial Intelligence–Enabled Clinical Algorithm Development: Formative Evaluation of the Semiautomatic Clinical Algorithm Development Framework
Source: JMIR Form Res. 2026 Mar 12;10:e90273. doi: 10.2196/90273 (PMC13022556; doi:10.2196/90273)
Supplement: Multimedia Appendix 2 [file formative_v10i1e90273_app2.docx]

**Analysis Objective:** To establish reliable medical facts about 'pediatric febrile seizures' by cross-validating information generated by four AI models (Consensus, ChatGPT, Gemini, Perplexity), and to lay the groundwork for the development of a clinical decision algorithm. This report is the output of Phase 2 of the S-ACAD workflow and served as the 'Single Source of Truth' in the subsequent algorithm development process.

**1. High Consensus**

This section contains key information that was consistently identified across all sources or is commonly recommended in major international guidelines.

- **Definition and Diagnostic Criteria**
  - A febrile seizure is a convulsion in a child aged 6 months to 5 years (60 months) that occurs with a fever of 38°C (100.4°F) or higher. [Confidence: Very High]
  - It is a diagnosis of exclusion, meaning there must be no history of central nervous system infection, metabolic disorders, or afebrile seizures. [Confidence: Very High]
  - It is the most common convulsive disorder in childhood, occurring in 2-5% of all children. [Confidence: Very High]
- **Classification Criteria**
  - Simple febrile seizure: Lasts less than 15 minutes, is generalized, and occurs once within a 24-hour period. [Confidence: Very High]
  - Complex febrile seizure: Meets one or more of the following: lasts 15 minutes or longer, is focal, or recurs within 24 hours. [Confidence: Very High]
  - Febrile Status Epilepticus: A seizure lasting 30 minutes or longer. [Confidence: High]
- **First Aid and Treatment Principles**
  - If a seizure lasts longer than 5 minutes, it is essential to call emergency medical services (911/119) immediately. [Confidence: Very High]
  - Lay the child on their side to secure the airway, do not put anything in their mouth, and do not forcibly restrain them. [Confidence: Very High]
  - Antipyretics are intended to relieve discomfort from fever and are not effective in preventing the recurrence of febrile seizures. [Confidence: Very High]
  - Daily or intermittent use of prophylactic anticonvulsants is not recommended due to the risk of side effects. [Confidence: Very High]
  - Tests such as EEG or brain imaging (CT/MRI) are not routinely necessary after a simple febrile seizure. [Confidence: High]
  - A lumbar puncture is performed selectively in cases with signs of meningitis or in unvaccinated infants aged 6-12 months. [Confidence: High]

**2. Conflicting Information**

This section details topics where criteria differ or conflict between sources or national guidelines.

- **Topic:** Diagnostic Criteria (Age and Fever)
  - **Claim A (AAP/NICE, etc. international standard):** 6 months to 5 years, 38.0°C or higher.
  - **Claim B (Korean Pediatric Neurological Society, etc.):** 3 months to 6 years, 37.8°C or higher.
  - **Analysis:** A clear discrepancy exists in the diagnostic criteria. For a global application, the international standard (e.g., AAP) should be the default, with local standards considered for regional localization.
- **Topic:** Prophylactic Diazepam Use
  - **Claim A (AAP/NICE):** Routine prophylactic use is not recommended due to the risk of side effects.
  - **Claim B (Japanese guidelines, etc.):** Intermittent use during fever is recommended for high-risk groups (e.g., complex type, family history).
  - **Analysis:** There is a clear difference in stance between Western and Eastern guidelines.
- **Topic:** Short-term Recurrence Prevention with Antipyretics
  - **Claim A (All guidelines):** No long-term recurrence prevention effect.
  - **Claim B (2021 Finnish RCT - single study):** Suggested the possibility of reducing short-term recurrence 'within the same febrile episode.'
  - **Analysis:** This is not a standard recommendation but is a controversial area that requires explanation during parent education.

**3. Unique Information & Needs Verification**

This section contains information mentioned only in limited sources or with low evidence levels, requiring further verification.

- **Complex Febrile Seizures and Long-term Prognosis:** Claims exist that the risk of ADHD is 1.4 times higher (OR 1.4) or IQ is 4.5 points lower on average in the group with a history of complex febrile seizures, but this has not been consistently confirmed in large-scale studies and is controversial. [Confidence: Medium]
- **Micronutrient (Iron, Zinc) Deficiency:** Some studies have reported a correlation suggesting that iron or zinc deficiency may be a risk factor, but causality is uncertain, and there is insufficient evidence for the preventive effect of supplementation. [Confidence: Low]
- **Use of Digital Education:** Information that some hospitals in Australia mandate text/video education before discharge is specific to certain institutions and difficult to generalize. [Confidence: Low]

**4. Overall Assessment and Conclusion**

- **Overall Reliability:** High. All sources show a high degree of agreement on core definitions, classifications, and first-aid principles.
- **Key Issues Proposed for Expert Validation:**
  - Standardization of Diagnostic Criteria
  - Guidelines for Prophylactic Anticonvulsant Use
  - Management Protocol for Infants Under 12 Months
  - Redefining the Role of Antipyretics
  - Inclusion of Uncertain Information on long-term prognosis.
