# Supplementary material for: Strategic Governance of Artificial Intelligence–Enabled Clinical Algorithm Development: Formative Evaluation of the Semiautomatic Clinical Algorithm Development Framework
Source: JMIR Form Res. 2026 Mar 12;10:e90273. doi: 10.2196/90273 (PMC13022556; doi:10.2196/90273)
Supplement: Multimedia Appendix 3 [file formative_v10i1e90273_app3.docx]

**Objective:** To establish a standardized response protocol to support caregiver decision-making and ensure clinical safety when a pediatric febrile seizure occurs. **Target Audience:** Global users, with a focus on the U.S. (units like temperature are dual-notated). **Evidence Level Notation Guide:**

- **Consensus:** The level of agreement among the multiple sources provided (A: High, B: Medium, C: Conservative Recommendation).
- **Evidence:** The type of medical evidence (Guideline, SR/Meta-analysis, Cohort/Observational Study, Expert Consensus/Review).

**Prerequisites (Input Data):** [Child's Name], [Age (in months)], [Underlying Conditions and Risk Factors (Boolean/Array)], [History of Febrile Seizures (Boolean)], [Prescribed Emergency Anticonvulsant (Boolean)], [Recent Vaccination History (String/Date)].

**START: Module Execution**

**[Decision 0: Initial Risk Factor Screening]**

- **[Question]** Does the child belong to any of the high-risk groups below?
  - Neurodevelopmental delay (e.g., cerebral palsy)
  - Direct family history of epilepsy
  - Other severe underlying conditions (e.g., congenital heart disease)
- **[Judgment]**
  - → **[Yes]**
    - FLAG: set high_risk = true.
    - **[Notification]** "[Child's Name] requires more careful observation due to an underlying condition. It is recommended to consult a doctor or visit the emergency room immediately if a seizure occurs."

Rationale: High-risk groups may have different causes and prognoses for seizures compared to non-high-risk groups, thus a more conservative approach (medical intervention) is induced from the start. [Consensus: A | Evidence: Cohort/Observational Study]

- - → **[No]**
    - FLAG: set high_risk = false.
    - **[Proceed]** Go to Decision 1

**[Decision 1: Immediate Top-Priority Emergency Filtering (Life-Threatening Red Flags)]**

- **[Question]** Does any of the following absolute emergency situations apply right now?
  - A. Is the child having difficulty breathing, or are their face/lips turning blue (cyanosis)?
  - B. Has the seizure already been going on for more than 5 minutes?
- **[Judgment]**
  - → **[Yes]** → **[Final Action]** 🔴 **Call 911 Immediately** (localize emergency number by country)

Rationale: A seizure lasting more than 5 minutes can progress to status epilepticus, and hypoxia is an emergency requiring immediate intervention. [Consensus: A | Evidence: Guideline]

- - → **[No]** → **[Proceed]** Go to Decision 2

**[Decision 2: Age Appropriateness Check]**

- **[Question]** Is the [Child's Age] between 6 months and 60 months (5 years) old?
- **[Judgment]**
  - → **[Under 6 months]** → **[Final Action]** 🔴 **Call 911 Immediately**

Rationale: Seizures in infants under 6 months have a very high risk of serious underlying conditions like meningitis. [Consensus: A | Evidence: Guideline]

- - → **[Over 60 months (5 years)]**
    - **[Additional Question]** Have they been diagnosed with a febrile seizure in the past?
    - **[Yes]** → **[Final Action]** 🟡 **Recommend ER Visit**
      - "Although over 5 years old, a history of febrile seizures warrants an ER visit to determine the cause. If the child is breathing stably and is clearly conscious after the seizure, you may consider going directly to the ER. However, if you are unsure or anxious, call 911 for advice."
    - **[No]** → **[Final Action]** 🔴 **Call 911 Immediately**

Rationale: A first seizure after the age of 5 is likely not a febrile seizure. [Consensus: A | Evidence: Guideline]

- - → **[6 to 60 months]** → **[Proceed]** Go to Decision 3

**[Decision 3: Fever and Vaccination Association Check]**

- **[Question 3-1]** Was there a 'fever' of ≥ 38.0°C (100.4°F) during the febrile illness at the time of or during the seizure?
  - → **[Definitely no (normal temperature)]** → **[Final Action]** 🔴 **Call 911 Immediately**

Rationale: An 'Afebrile Seizure' requires immediate neurological evaluation. [Consensus: A | Evidence: Guideline]

- - → **[Couldn't measure/Uncertain]**
    - **[Additional Question]** Did the child's body feel hot before or after the seizure?
    - **[Yes]** → **[Proceed]** Go to Decision 3-2
    - **[No]** → **[Final Action]** 🟡 **Contact Medical Facility in Steps**
      - "Since it's uncertain whether there was a fever, an accurate evaluation is needed. Please follow the steps below."
      - "Step 1: First, try to contact your primary care physician's (PCP) after-hours on-call line within 5 minutes."
      - "Step 2: If you cannot reach them, or if you are advised to go to the ER immediately, go to the ER right away."
  - → **[Yes (38.0°C or higher)]** → **[Proceed]** Go to Decision 3-2
- **[Question 3-2]** Has the child been vaccinated within the last 48 hours?
  - → **[Yes]**
    - **[Information]** "A seizure can be triggered by a fever that occurs after vaccination. This is a reaction to the fever, not the vaccine itself, and most cases have a good prognosis. This information is very important for the medical staff, so please be sure to tell them." [Consensus: A | Evidence: Observational Study/Review]
    - **[Proceed]** Execute [Situation Protocol: Seizure in Progress]
  - → **[No]**
    - (No information provided)
    - **[Proceed]** Execute [Situation Protocol: Seizure in Progress]

**[Situation Protocol: Seizure in Progress]**

- **[Action 1]** ✅ **Top Priority Safety Measures:** Lay the child on their side and clear the surrounding area. [Consensus: A | Evidence: Guideline]
- **[Action 2]** ⏱️ **Time and Observe:** Start a timer and observe the seizure's characteristics (record a video if possible). [Consensus: A | Evidence: Expert Consensus]
- **[Action 3]** ❌ **Absolute Don'ts:** Do not put anything in the mouth, do not forcibly restrain, etc. [Consensus: A | Evidence: Guideline]

**[Decision 4: Response if Seizure Lasts 5 Minutes]**

- **[Question]** Has it been 5 minutes since the seizure started?
  - → **[No]** → **[Proceed]** Wait until the seizure stops
  - → **[Yes]**
    - **[Question 4-1]** Do you have an emergency anticonvulsant (e.g., rectal Diazepam) prescribed by a doctor?
    - **[No]** → **[Final Action]** 🔴 **Call 911 Immediately**
    - **[Yes]** → **[Final Action]** 🔴 **Call 911 Immediately** and say the following:
      - "My child has been seizing for more than 5 minutes, and I have an emergency medication (like Diazepam) prescribed by a doctor. Please give me instructions on its administration."

Rationale: The use of emergency medication is safest when done under the real-time guidance of a 911 dispatcher or medical professional. [Consensus: A | Evidence: Expert Consensus/Guideline]

**[Situation Protocol: Seizure Stopped]**

**[Decision 5: Post-ictal State Evaluation]**

- **[Question 5-1]** Is the child breathing normally?
  - → **[No]** → **[Final Action]** 🔴 **Call 911 Immediately**
- **[Question 5-2]** What is the child's level of consciousness? (Apply AVPU Scale)
  - A (Alert): Awake, aware of surroundings, and makes eye contact with parents?
  - V (Verbal): Responds to speech (babbling) or sounds?
  - P (Pain): Responds only to mild stimuli (like a pinch)?
  - U (Unresponsive): No response to any stimuli?
- **[Judgment]**
  - → **[P or U state]** → **[Final Action]** 🔴 **Call 911 Immediately**
  - → **[V state continues for >10 mins]** → **[Final Action]** 🟡 **Visit the ER**
    - "A slow recovery of consciousness after a seizure requires an ER visit. If the child is gradually improving and breathing is stable, you can go directly to the ER. However, if they worsen at all or you are anxious, call 911 immediately."
  - → **[A or V state (improving)]** → **[Proceed]** Go to Decision 6

Rationale: Delayed recovery of consciousness after a seizure may indicate a serious underlying condition. The AVPU scale is a tool that caregivers can use for objective assessment. [Consensus: A | Evidence: Guideline]

**[Decision 6: Determining the Level of Hospital Visit]**

- **[Question]** The seizure has stopped, and the child is stabilizing. Which of the following applies?
  - **A. Cases Requiring an Immediate ER Visit:**
    - [ ] Seizure lasted more than 15 minutes (complex febrile seizure)
    - [ ] Focal seizure (e.g., only one arm/leg shaking) (complex febrile seizure)
    - [ ] Seizure occurred more than once within 24 hours (complex febrile seizure)
    - [ ] Infant 6-12 months old + incomplete Hib/pneumococcal vaccination
    - [ ] Incomplete recovery of consciousness or child seems very lethargic after the seizure
    - [ ] high_risk flag is true (underlying condition)
  - **B. Cases Requiring a Same-Day Visit (Urgent Care or PCP):**
    - [ ] First-ever febrile seizure (with full recovery afterward)
    - [ ] Parent is worried about the child's condition and wants medical consultation
  - **C. Cases Where a Next-Day PCP Visit is Possible:**
    - [ ] Previously diagnosed with a simple febrile seizure, the current episode was similar, and the child has fully returned to their usual condition afterward
    - [ ] Parent fully understands the child's condition and feels reassured
- **[Judgment]**
  - → **[Any item in A applies]** → **[Final Action]** 🟡 **Visit the ER**
    - "The features mentioned suggest a 'complex febrile seizure' or the possibility that other causes need to be ruled out. Please go to the ER now for an evaluation."
  - → **[Applies to B or C]** → **[Final Action]** ✅ **Check Time-Based Care Guidance**
    - "The child's condition appears stable. Please choose the most appropriate medical facility based on the current time."
    - **[Weekdays (8 AM - 6 PM)]**
      - If B applies (Same-Day Visit): 🟢 Contact a nearby PCP or Urgent Care for a visit. This may be faster and more economical.
      - If C applies (Next-Day Visit): 🔵 Contact your PCP to inform them of the seizure and schedule a visit for the next day.
    - **[Nights / Weekends]**
      - If B or C applies: 🟢 The best first step is to contact your PCP's on-call line for advice. If that's difficult, check for pediatric Urgent Care centers that are open. If neither of these options is feasible, you should consider visiting the ER.
- 💡 **Insurance & Accessibility Note**
  - **Cost Consideration:** In general, medical costs are highest at the ER, followed by Urgent Care, and then the PCP. Insurance coverage may vary depending on your individual plan.
  - **Regional Differences:** Depending on your location (e.g., urban vs. rural), there may be no available Urgent Care, or ER wait times could be very long. Please make your decision considering these practical constraints.
- ✅ **Post-Consultation Follow-Up**
  - After receiving medical care for the seizure, please check the following to continue managing your child's health.
  - [ ] **Share Records:** If you visited an Urgent Care or ER, be sure to forward the medical records to your child's PCP.
  - [ ] **Vaccination Plan:** If any vaccinations were delayed due to the seizure, consult with your PCP to reschedule them safely.
  - [ ] **Recurrence Action Plan:** In case a seizure happens again, create an action plan in advance with your PCP, including the potential need for a prescription for an emergency anticonvulsant.
- **[Common Safety Alert]**
  - ⚠️ **Call 911 or visit the ER immediately if any of the following occur:**
    - Another seizure occurs
    - Consciousness worsens
    - Persistent vomiting
    - Stiff neck
    - Seems to have difficulty breathing
    - Your parental intuition tells you something is wrong
  - 💡 When in doubt, always choose a higher level of care. Your child's safety is the top priority.
