# Supplementary material for: Strategic Governance of Artificial Intelligence–Enabled Clinical Algorithm Development: Formative Evaluation of the Semiautomatic Clinical Algorithm Development Framework
Source: JMIR Form Res. 2026 Mar 12;10:e90273. doi: 10.2196/90273 (PMC13022556; doi:10.2196/90273)
Supplement: Multimedia Appendix 4 [file formative_v10i1e90273_app4.docx]

This appendix provides a detailed record of the *AI Sparring* and iterative improvement process, which is the core of the S-ACAD workflow. It shows, step-by-step, how the initial AI-generated draft was progressively refined through feedback from an AI critic and a human expert to become the final algorithm (v1.0). The quantitative data described in section 3.4 of the main text, 'AI Sparring Effectiveness Analysis,' is based on the detailed analysis below.

**Iteration 1:** From Initial Draft (v0.1) to User-Centric Design (v2.0)

- **Step 1.1:** Initial Draft Generation (v0.1 by Gemini)
  - **Input:** 4 foundational data documents + 1 synthesis report
  - **Prompt:** See Multimedia Appendix 1 - Request to generate an evidence-based algorithm draft for parents.
  - **Output (v0.1 Summary):** Medically accurate, but a text-heavy, list-based structure. It was divided into [Before Seizure], [During Seizure], and [After Seizure], with Red Flag conditions scattered throughout.
- **Step 1.2:** Critical Review (by Claude) & Quantitative Analysis
  - **Input:** Algorithm v0.1
  - **Prompt:** Request for a multi-faceted critique covering logical flow, user experience, safety, and edge cases.
  - **Output (Summary of 16 Suggestions):** The Claude model identified a total of 16 significant improvement suggestions. After review by a human expert, 14 of these (87.5%) were adopted into the final algorithm.

Table S1: Review and Implementation Results of AI-Generated Suggestions

| **Category** | **Suggestion Content** | **Adoption Status** | **Rationale and Outcome** |
| --- | --- | --- | --- |
| **Logical Flaws (3/3)** | 1. Confusion in 119 call timing (mixing 5-minute standard and immediate call conditions) | Adopted (O) | Starting from v2.0, an 'Immediate 911' section was placed at the very top to clarify priority. |
|  | 2. Ambiguity in priority between 119 call for first seizure and hospital visit guidelines | Adopted (O) | From v2.0, 'first seizure' was elevated to an 'Immediate 911' condition, then clarified to 'ER visit' after the seizure. |
|  | 3. Omission of exception rules for infants under 6 months (mentioned in body) | Adopted (O) | Explicitly included in the age filter logic from v3.1. |
| **UX Improvement (5/7)** | 4. Use of specialized terms like "complex febrile seizure" (mentioned in body) | Adopted (O) | Replaced with simple explanations like "lasts more than 15 minutes..." from v2.0. |
|  | 5. Use of non-intuitive terms like "photosensitivity" | Adopted (O) | Changed to "cries excessively or avoids bright lights" from v2.0. |
|  | 6. Presentation of unmeasurable criteria like "when the neck is stiff" | Adopted (O) | Specified as "when the chin cannot touch the chest" from v2.0. |
|  | 7. Text-heavy structure reduces readability in urgent situations | Adopted (O) | Icon system (🚨, ✅, etc.) introduced from v2.0. |
|  | 8. Equal visual importance of DOs and DON'Ts makes priority identification difficult | Adopted (O) | Changed to a format that emphasizes 'Top Priority Safety Actions' from v2.0. |
|  | 9. All text in black only makes it difficult to distinguish urgency | Not Adopted (X) | Judged that the icon system adequately expresses urgency. |
|  | 10. Entire algorithm length is long, leading to severe scroll pressure (accordion menu suggestion) | Not Adopted (X) | Judged that having all information expanded is safer in an emergency. |
| **Missing Scenarios (4/4)** | 11. Handling cases where a child falls into a deep sleep after a seizure and is difficult to wake (mentioned in body) | Adopted (O) | Included in the 'Consciousness Check' step with the criterion "if they don't open their eyes after 10 minutes" from v2.0. |
|  | 12. Response protocol for seizures occurring during vehicle travel | Adopted (O) | Included in 'Special Situation Management' from v4.0. |
|  | 13. Response protocol for seizures occurring during bathing | Adopted (O) | Included in 'Special Situation Management' from v4.0. |
|  | 14. Response protocol for seizures occurring in public places | Adopted (O) | Included in 'Special Situation Management' from v4.0. |
| **Safety Enhancement (2/2)** | 15. Insufficient emphasis on not putting anything in the mouth during a seizure (mentioned in body) | Adopted (O) | Strongly warned with a ❌ icon in the 'Absolute Don'ts' section from v2.0. |
|  | 16. Failure to reflect the practical difficulty of time measurement (suggestion for 119 dispatcher assistance) | Adopted (O) | Added the phrase "Call 911 and ask the operator to help you keep time" from v2.0. |

- **Step 1.3: Revision and Output (v2.0)**
  - **Action:** Fully adopted Claude's critique to redesign the algorithm with a focus on user experience (UX).
  - **Key Changes in v2.0:**
    - **Structure Overhaul:** Placed absolute situations requiring an 🚨 immediate 911 call at the very top to clarify priority.
    - **Visualization Introduced:** Implemented an icon system: 🚨 (Immediate 911), ✅ (Safety Action), ⏱️ (Time Check), ❌ (Absolute Don't).
    - **Simplification:** Simplified the complex [After Seizure] decision-making into three steps: Breathing → Consciousness → Hospital Visit Decision.
    - **Terminology Improvement:** Changed to measurable and easy expressions like "when the chin cannot touch the chest."

**Iteration 2: Personalization and Contextualization (v2.0 -> v3.1)**

- **Step 2.1: New Requirements (by Human Expert)**
  - **Feedback:** "Since this is a module within the Fevercoach app, it must utilize pre-saved user information (age, history, etc.). Specifically, the definition of a febrile seizure (6-60 months, accompanied by fever) must be clearly filtered at the start of the algorithm."
- **Step 2.2: Revision and Output (v3.1)**
  - **Action:** Added personalization logic using pre-saved information and a core definition filter.
  - **Key Changes in v3.1:**
    - **Personalization:** Dynamically displays the child's name, as in "A personalized guide for [Child's Name]'s parents."
    - **Age Filter Added (Decision 1):** Checks the 6-60 month range at the start of the algorithm, setting a path to call 911 immediately if outside this range.
    - **Fever Filter Added (Decision 2):** Checks if there was a 'fever' during the seizure, branching to an immediate 911 call for 'afebrile seizures' without fever.

**Iteration 3: Expert Review and Globalization (v3.1 -> v4.2)**

- **Step 3.1: Expert-level Review**
  - **Input:** Algorithm v3.1
  - **Feedback:** "As a global app, it must follow international standards (AAP). The temperature criteria, consciousness recovery assessment criteria, etc., need to be refined more precisely, and screening for high-risk groups like those with underlying conditions is necessary."
- **Step 3.2: Revision and Output (v4.2)**
  - **Action:** Reflected expert feedback to enhance clinical precision and compliance with global standards.
  - **Key Changes in v4.2:**
    - **Global Standard Applied:** Temperature criterion corrected to 38.0°C (100.4°F).
    - **Risk Stratification (Decision 0):** Added an initial risk factor screening step to identify high-risk groups beforehand.
    - **Objective Assessment Tool Introduced:** Applied the AVPU Scale for consciousness recovery assessment.
    - **Observation Items Detailed:** Specified seizure characteristics such as Tonic/Clonic/Atonic, bilateral/unilateral, etc.

**Iteration 4: Final Safety Enhancements and Nuanced Guidance (v4.2 -> v1.0 Final)**

- **Step 4.1: Final Safety & Usability Check (by Human Expert)**
  - **Input:** Algorithm v4.2
  - **Feedback:** "The association with vaccination must be specified, and the protocol for using prescribed emergency anticonvulsants must be modified in the safest way possible. Also, rather than having all situations result in a 'Call 911,' the guidance should be segmented into 'ER Visit,' 'Same-Day Visit,' etc., based on the urgency of the situation, to promote the efficient use of medical resources and reduce the user's burden."
- **Step 4.2: Revision and Finalization (v1.0)**
  - **Action:** Completed the algorithm by reflecting final safety and usability review comments.
  - **Key Changes in v1.0:**
    - **Vaccination History Added (Decision 3.2):** Checks for recent vaccinations and guides the user to report this information to medical staff.
    - **Emergency Medication Protocol Modified (Decision 4):** Instead of the app directly instructing medication administration, the path was modified to 🚨 call 911 immediately, inform them of the medication possession, and follow the medical professional's instructions, minimizing legal/safety risks.
    - **Hospital Visit Level Segmented (Decision 6):** For patients stable after a seizure, the guidance was segmented into ER visit, same-day visit, and next-day visit, with customized guidance for different times (day/night/weekend) to increase practicality.
    - **Evidence Level Notation Completed:** Added dual [Consensus | Evidence] notation and citations to all decision points.

Through this multi-stage process of iterative validation and refinement, the initial idea was able to evolve into a final algorithm (v1.0) that meets the complexity and safety requirements of the actual clinical setting.
