# Supplementary material for: Strategic Governance of Artificial Intelligence–Enabled Clinical Algorithm Development: Formative Evaluation of the Semiautomatic Clinical Algorithm Development Framework
Source: JMIR Form Res. 2026 Mar 12;10:e90273. doi: 10.2196/90273 (PMC13022556; doi:10.2196/90273)
Supplement: Multimedia Appendix 5 [file formative_v10i1e90273_app5.docx]

F-ACAD represents an experimental baseline configuration constructed on a specific platform (Genspark) to illustrate the risks of ungoverned automation, rather than a definitive representation of all fully automated approaches.

**A5.1 F-ACAD System Architecture**

**Overview**

The F-ACAD (Fully Autonomous Clinical Algorithm Development) system consists of 14 specialized AI agents operating autonomously to develop clinical algorithms without human intervention beyond initial task specification and final review.

**Agent Configuration**

1. Search Agents (4):

- US Guideline Specialist: Focuses on AAP, ACEP guidelines

- International Guideline Specialist: NICE, ILAE, global standards

- Systematic Review Specialist: Cochrane, PubMed meta-analyses

- Emerging Research Specialist: Recent studies (2020-2025), controversial topics

2. Validation & Synthesis Agents (3):

- Verification Agent: Source and reference validation

- Synthesis Agent: Data integration and guideline comparison

- Quality Control Agent: Consistency and completeness review

3. Design Agents (2):

- Algorithm Design Agent: Decision tree generation

- Content Generation Agent: Parent-friendly educational modules

4. Critic Agents (4):

- US Pediatrician AI: Clinical accuracy and standard compliance

- Pediatric Emergency AI: Emergency classification and response

- Parent UX AI: Language clarity and user experience

- Medical Informatics AI: Logical completeness and data structure

5. Improvement Management Agent (1):

- Prioritizes critiques and applies improvements iteratively

**A5.2 Detailed Time Logs Comparison**

**Table S1.** Phase-by-Phase Time Comparison Between S-ACAD and F-ACAD (Single-Run Operational Estimates)

| **Development Phase** | **S-ACAD Time (single-run estimate)** | **F-ACAD Time (single-run estimate)** | **Relative difference (illustrative)** |
| --- | --- | --- | --- |
| Phase 1: Data Collection | ≈30 min | ≈10 min 22 sec | 65.4% |
| Phase 2: Validation & Synthesis | ≈125 min | ≈9 min 33 sec | 92.4% |
| Phase 3: Algorithm Generation and Refinement | ≈60 min | ≈18 min 03 sec* | 69.9% |
| Phase 4: Final Expert Review | ≈30 min | ≈30 min** | 0% |
| **Total** | **245 min** | **67 min 58 sec** | **72.3%** |

General note: All time values (including those reported for S-ACAD) represent approximate, single-run operational estimates derived from one end-to-end execution and are provided to characterize relative workflow burden rather than statistically representative performance benchmarks.

* For F-ACAD, ‘Algorithm Generation’ (10 min 15 sec) and ‘Iterative Refinement’ (7 min 48 sec) are aggregated to align with S-ACAD’s Phase 3 (‘Algorithm Generation and Refinement’) for an illustrative phase-level comparison.

** For F-ACAD, ‘Final Expert Review’ time is assumed to match S-ACAD’s Phase 4, reflecting the second human touchpoint described in the methodology; this assumption is used solely to support workflow characterization.

**A5.3 AI Agent Specifications and Performance**

**Table S2.** F-ACAD Agent Activity Log

| **Agent Category** | **Number of Agents** | **Total API Calls** | **Processing Time** |
| --- | --- | --- | --- |
| Search Agents | 4 | 47 | 10 min 22 sec |
| Validation Agents | 3 | 23 | 9 min 33 sec |
| Design Agents | 2 | 18 | 10 min 15 sec |
| Critic Agents | 4 | 31 | 7 min 48 sec |
| Management Agent | 1 | 12 | Throughout |

**A5.4 Comprehensive S-ACAD vs F-ACAD Comparison**

**Table S3.** Detailed Methodology Comparison

| **Aspect** | **S-ACAD** | **F-ACAD** |
| --- | --- | --- |
| **Process Control** | Human-guided at each phase | Fully autonomous |
| **Decision Points** | 19 human interventions | 0 (post-initial prompt) |
| **Quality Assurance** | Human expert validation | AI sparring validation |
| **Error Detection** | Human clinical judgment | Multi-agent critique |
| **Contextual Understanding** | Deep, nuanced | Surface-level |
| **Safety Considerations** | Proactive, comprehensive | Reactive, rule-based |
| **Adaptability** | High (human insight) | Limited (predefined logic) |

**A5.5 F-ACAD AI Sparring Results**

**Critical Issues Identified by AI Critics (Total: 17)**

**High Priority (9):**

1. Inconsistent emergency response timing (3-5 minute ambiguity)
2. Missing U.S. liability considerations
3. Inadequate differentiation between ER vs. PCP visits
4. Complex medical terminology without lay explanations
5. Insufficient empathetic tone for distressed parents
6. Medication dosing guidelines too generic
7. Missing age-specific nuances for <12 months
8. Incomplete post-seizure monitoring guidance
9. Structural redundancy in decision nodes

**Medium Priority (8):**

1. Inconsistent terminology (epilepsy vs. seizure disorder)
2. Limited visual guidance references
3. Missing vaccination history integration
4. Unclear time-of-day considerations
5. Insufficient multi-language considerations
6. Limited caregiver type differentiation
7. Missing insurance/cost guidance
8. Incomplete follow-up timeline specifications

**A5.6 Key Insights from Comparison**

**Where F-ACAD Excelled:**

- Rapid parallel data collection
- Comprehensive source coverage (25 vs. 18 sources)
- Consistent structure generation
- Automated cross-validation

**Where S-ACAD Proved Superior:**

- Clinical judgment integration
- Safety-first approach
- Contextual nuance capture
- Parent-centric communication
- Liability and ethical considerations

**Convergence Analysis:** Both approaches produced plausible algorithm drafts. However, the quality gap became evident during AI sparring: F-ACAD generated 17 critical issues (including 9 high-priority safety/accuracy concerns) requiring correction, whereas many of these issues were addressed earlier through 19 human expert interventions in S-ACAD. Overall, S-ACAD took ~3.6× longer than F-ACAD (245 min vs 67 min 58 sec), but it incorporated continuous expert validation and an independent clinical review checkpoint prior to any deployment. This was consistent with external safety verification reporting no medically inaccurate sections or critical safety errors requiring mandatory correction.

**A5.7 Recommended Hybrid Approach**

Based on the comparison, an optimal hybrid approach would:

1. Use F-ACAD for Phase 1 (Data Collection) - 10 minutes
2. Apply human review at Phase 2 (Validation) - 30 minutes
3. Use F-ACAD for Phase 3 (Draft Generation) - 10 minutes
4. Apply human-guided refinement at Phase 4 - 30 minutes
5. Automate Phase 5 (Documentation) - 9 minutes

**Estimated Total Time: ~90 minutes** (63% reduction from S-ACAD, with quality preservation)
