# Supplementary material for: Strategic Governance of Artificial Intelligence–Enabled Clinical Algorithm Development: Formative Evaluation of the Semiautomatic Clinical Algorithm Development Framework
Source: JMIR Form Res. 2026 Mar 12;10:e90273. doi: 10.2196/90273 (PMC13022556; doi:10.2196/90273)
Supplement: Multimedia Appendix 6 [file formative_v10i1e90273_app6.docx]

**External Expert Review Form: [Pediatric Febrile Seizure Response Algorithm]**

**1. Overview of the Review Request**

- **Research Title:** Rapid Development of Parent-Actionable Pediatric Febrile Seizure Guidance Using a Human-AI Collaborative Workflow (S-ACAD): A Prospective Proof-of-Concept Study
- **Purpose of Review:** This document aims to systematically verify the clinical validity, safety, and completeness of the 'Pediatric Febrile Seizure Response Algorithm (v1.0),' which was rapidly developed through human-AI collaboration, from an independent, external expert's perspective.
- **Review Materials:** Pediatric Febrile Seizure Response Algorithm (v1.0) text, with the flowchart diagram attached separately.
- **Use of Review Results:** Your review comments will be **anonymized** and utilized as quantitative and qualitative data to substantiate the reliability of this algorithm and ensure the objectivity of the manuscript.
- **Contact:** Sang-Hyun Ahn (ansang2@naver.com)

Thank you for taking your valuable time to provide this expert review.

**2. Reviewer Information**

- **Name:** _______________
- **Affiliation:** _______________
- **Specialty:** Board-Certified Pediatrician (Yes/No) / **Subspecialty:** ___________
- **Clinical Experience:** Total _____ years
- **Date of Review:** _____ / _____ / 2025

**Part A: Quantitative Validity Assessment**

Please rate your agreement with each item on a 5-point scale.

(1: Strongly Disagree, 2: Disagree, 3: Neutral, 4: Agree, 5: Strongly Agree)

| Assessment Domain | Detailed Assessment Item | Scale (1-5) |
| --- | --- | --- |
| **1. Clinical Accuracy** | 1-1. The algorithm's decision-making criteria are consistent with current clinical guidelines and medical evidence. | 1—2—3—4—5 |
|  | 1-2. The Red Flag symptoms for identifying emergencies are clinically valid and appropriately included. | 1—2—3—4—5 |
|  | 1-3. The recommendations at each step (e.g., first aid, tests) are medically accurate. | 1—2—3—4—5 |
| **2. Completeness** | 2-1. Most major clinical scenarios that a caregiver might experience (e.g., first seizure, recurrence) are included. | 1—2—3—4—5 |
|  | 2-2. Exceptional situations (e.g., under 6 months, over 5 years) are appropriately handled. | 1—2—3—4—5 |
|  | 2-3. There are no apparent missing steps or logical flaws in the decision-making pathways. | 1—2—3—4—5 |
| **3. Safety** | 3-1. The criteria for identifying life-threatening situations are appropriately placed with the highest priority. | 1—2—3—4—5 |
|  | 3-2. The response guidelines for potentially dangerous situations are sufficiently conservative and safely designed. | 1—2—3—4—5 |
|  | 3-3. Safeguards to prevent potential harm (e.g., warnings for prohibited actions) are effectively included. | 1—2—3—4—5 |
| **4. Usability for Parents** | 4-1. Medical terms are explained in simple language that a caregiver can understand. | 1—2—3—4—5 |
|  | 4-2. The action guidelines are clear and practically executable in an emergency. | 1—2—3—4—5 |
|  | 4-3. The overall decision-making flow is logical and easy to follow. | 1—2—3—4—5 |

**Part B: Qualitative In-depth Review**

We would appreciate your in-depth expert opinion. Please describe freely.

B-1. What do you consider to be the greatest strength of this algorithm?

_____________________________________________________________________________

**B-2. Are there any parts that you believe are medically inaccurate or potentially misleading and require mandatory correction? (If yes, please specify the node number and suggest a revision.)**

( ) None

( ) Yes (Details below):

Node #___: ________________________________________________________________

Node #___: ________________________________________________________________

**B-3. Are there any important clinical scenarios or Red Flag symptoms missing from the current algorithm? (If yes, please specify.)**

( ) None

( ) Yes (Details below):

_____________________________________________________________________________

**Part C: Overall Assessment & Final Recommendation**

**C-1. Please rate the overall clinical validity of this algorithm on a scale of 1 to 10.**

Overall Score: _____ / 10

**C-2. Compared to existing written/verbal educational materials for parents, how would you rate the usefulness of this algorithm?**

( ) Much Better

( ) Somewhat Better

( ) About the Same

( ) Somewhat Worse

( ) Much Worse

**C-3. Assuming it successfully passes usability testing with caregivers, what is your opinion on using this algorithm for the development of an actual parent-facing service?**

( ) Usable in its current state

( ) Usable after minor revisions

( ) Requires major revisions

( ) Not suitable for use

**C-4. If you have any other comments or suggestions, please describe them freely.**

_____________________________________________________________________________

Thank you again for providing your expert review.
