# Supplementary material for: Strategic Governance of Artificial Intelligence–Enabled Clinical Algorithm Development: Formative Evaluation of the Semiautomatic Clinical Algorithm Development Framework
Source: JMIR Form Res. 2026 Mar 12;10:e90273. doi: 10.2196/90273 (PMC13022556; doi:10.2196/90273)
Supplement: Multimedia Appendix 7 [file formative_v10i1e90273_app7.docx]

This appendix contains the full, anonymized text of the qualitative feedback (Parts B and C) provided by the two independent external reviewers. The original responses in Korean were translated into English by the author.

**Reviewer A (Pediatric Emergency Physician, 15 years of experience)**

**B-1. What do you consider to be the greatest strength of this algorithm?**

"It provides comprehensive and accurate guidance for febrile seizures, which can be relatively common in children, especially for situations that require attention."

**B-2. Are there any parts that require mandatory correction?**

None.

**B-3. Are there any important clinical scenarios or Red Flag symptoms missing?**

None.

**C-4. Other comments or suggestions?**

"Although the differences in medical access and delivery systems between the U.S. and South Korea must be considered, I have doubts about whether a non-medical caregiver can simply observe a convulsing child for more than 5 minutes without taking special measures."

**Reviewer B (Primary Care Pediatrician, 15 years of experience)**

**B-1. What do you consider to be the greatest strength of this algorithm?**

"When a child has a seizure at home, it provides parents with a way to respond, allowing them to observe the patient safely, which can likely provide reassurance to the caregiver."

**B-2. Are there any parts that require mandatory correction?**

None.

**B-3. Are there any important clinical scenarios or Red Flag symptoms missing?**

None.

**C-4. Other comments or suggestions?**

*(No comments provided)*
