# Supplementary material for: Strategic Governance of Artificial Intelligence–Enabled Clinical Algorithm Development: Formative Evaluation of the Semiautomatic Clinical Algorithm Development Framework
Source: JMIR Form Res. 2026 Mar 12;10:e90273. doi: 10.2196/90273 (PMC13022556; doi:10.2196/90273)
Supplement: Multimedia Appendix 8 [file formative_v10i1e90273_app8.docx]

This appendix provides the itemized, anonymized quantitative scores from the two independent external reviewers for transparency. These raw data supplement the qualitative findings and safety verification discussed in the main manuscript's Results section. The evaluation instrument can be found in Multimedia Appendix 6.

**Disclaimer:** Due to the small sample size (N=2), these scores lack the statistical power required for generalization. They should be interpreted primarily as qualitative indicators within this proof-of-concept study, rather than statistically validated metrics.

**Part A: Quantitative Validity Assessment (5-Point Likert Scale)**

| Assessment Domain | Detailed Assessment Item | Reviewer A Score | Reviewer B Score |
| --- | --- | --- | --- |
| **1. Clinical Accuracy** | 1-1. Consistency with clinical guidelines and evidence. | 5 | 5 |
|  | 1-2. Validity of Red Flag symptoms for emergencies. | 5 | 5 |
|  | 1-3. Medical accuracy of recommendations. | 5 | 5 |
|  | **Domain Average** | **5.0** | **5.0** |
| **2. Completeness** | 2-1. Inclusion of major clinical scenarios. | 5 | 5 |
|  | 2-2. Appropriate handling of exceptional situations. | 5 | 4 |
|  | 2-3. Absence of missing steps or logical flaws. | 5 | 5 |
|  | **Domain Average** | **5.0** | **4.67** |
| **3. Safety** | 3-1. Appropriate prioritization of life-threatening situations. | 5 | 5 |
|  | 3-2. Conservative and safe design of guidelines. | 5 | 5 |
|  | 3-3. Effective inclusion of safeguards and warnings. | 5 | 5 |
|  | **Domain Average** | **5.0** | **5.0** |
| **4. Usability for Parents** | 4-1. Simple language and clear explanation of terms. | 5 | 5 |
|  | 4-2. Practical executability of action guidelines. | **3** | 5 |
|  | 4-3. Logical and easy-to-follow decision-making flow. | 5 | 5 |
|  | **Domain Average** | **4.33** | **5.0** |

*Note: The domain averages presented in the main manuscript (e.g., Completeness: 4.8/5.0, Usability: 4.7/5.0) are the rounded averages of the two reviewers' domain-specific average scores.*

**Part C: Overall Assessment & Final Recommendation**

| Assessment Item | Reviewer A (Emergency Physician) | Reviewer B (Primary Care Pediatrician) |
| --- | --- | --- |
| **C-1. Overall Clinical Validity (out of 10)** | 9.0 / 10 | 9.5 / 10 |
| **C-2. Usefulness vs. Existing Materials** | Much Better | Much Better |
| **C-3. Recommendation for Actual Service** | Usable after minor revisions | Usable after minor revisions |

*Note: The average overall clinical validity score reported in the manuscript (9.25/10) is the mean of the two reviewers' scores.*

**Multimedia Appendix 9:** AI Sparring Effectiveness and Human Intervention Analysis

This appendix presents the visual analysis of the AI Sparring process and human expert interventions recorded during the S-ACAD workflow, which was moved from the main manuscript to comply with JMIR style requirements prohibiting pie charts and histograms in the main text.

**Panel (A): AI sparring effectiveness analysis.** The bar chart compares the number of issues identified by the AI critic (Claude) during Phase 3 against the number subsequently adopted into the final algorithm after expert review. Categories include UX improvements (7 identified, 5 adopted), missing scenarios (4 identified, 4 adopted), logic gaps (3 identified, 3 adopted), and safety enhancements (2 identified, 2 adopted). Of the 16 total suggestions, 14 (14/16, 87.5%) were adopted.

**Panel (B): Distribution of human interventions by type (total N=19).** The donut chart shows the distribution of all 19 human expert interventions recorded across the S-ACAD workflow. Clinical judgment was the most frequent category (8/19, 42.1%), followed by safety review (5/19, 26.3%), UX optimization (3/19, 15.8%), prompt adjustment (2/19, 10.5%), and other (1/19, 5.3%). Note: The categories were retrospectively assigned by the first author (SHA) without independent verification; these proportions should be interpreted as exploratory rather than validated measurements.

*Note: The corresponding figure image is uploaded separately in the submission system.*
