# Supplementary material for: Strategic Governance of Artificial Intelligence–Enabled Clinical Algorithm Development: Formative Evaluation of the Semiautomatic Clinical Algorithm Development Framework
Source: JMIR Form Res. 2026 Mar 12;10:e90273. doi: 10.2196/90273 (PMC13022556; doi:10.2196/90273)
Supplement: Multimedia Appendix 9 [file formative_v10i1e90273_app9.pdf]

SQUIRE 2.0 Reporting Checklist

Standards for QUality Improvement Reporting Excellence

Manuscript #90273: Strategic Governance of AI-Enabled Clinical Algorithm Development: A Formative Evaluation of the S-ACAD Framework

Ogrinc G, Davies L, Goodman D, Batalden PB, Davidoff F, Stevens D. SQUIRE 2.0 (Standards for QUality Improvement Reporting Excellence): revised publication guidelines from a detailed consensus process. BMJ Qual Saf 2016;25(12):986–992. doi: 10.1136/bmjqs-2015-004411 PMID:26369893

SQUIRE Item	Description	Reported on Page/Section	Comment
**Title and Abstract**			
**1. Title**	Indicate that the manuscript concerns an initiative to improve healthcare (broadly defined to include quality, safety, effectiveness, patient-centeredness, timeliness, cost, efficiency, and equity of healthcare).	Title, p. 1	*Yes*
**2. Abstract**	a. Provide adequate information to aid in searching and indexing. b. Summarize all key information from various sections of the text using the abstract format of the intended publication.	Abstract, p. 1	*Yes*
**Introduction — Why did you start?**			
**3. Problem Description**	Nature and significance of the local problem.	Section 1, ¶1–2	*Yes*
**4. Available Knowledge**	Summary of what is currently known about the problem, including relevant previous studies.	Section 1, ¶3–5	*Yes*
**5. Rationale**	Informal or formal frameworks, models, concepts, and/or theories used to explain the problem, any reasons or assumptions that were used to develop the intervention(s), and reasons why the intervention(s) was expected to work.	Section 1, ¶6–7	*Yes*
**6. Specific Aims**	Purpose of the project and of this report.	Section 1, final ¶	*Yes*
**Methods — What did you do?**			
**7. Context**	Contextual elements considered important at the outset of introducing the intervention(s).	Section 2, ¶1; Section 2.9	*Yes*
**8. Intervention(s)**	a. Description of the intervention(s) in sufficient detail that others could reproduce it. b. Specifics of the team involved in the work.	Sections 2.1–2.4; Multimedia Appendices 1–4	*Yes*
**9. Study of the Intervention(s)**	a. Approach chosen for assessing the impact of the intervention(s). b. Approach used to establish whether the observed outcomes were due to the intervention(s).	Sections 2.5–2.7	*Yes*
**10. Measures**	a. Measures chosen for studying processes and outcomes of the intervention(s), including rationale for choosing them. b. Description of the approach to the ongoing assessment of contextual elements. c. Methods employed for assessing completeness and accuracy of data.	Section 2.5 (metrics); Section 2.6 (expert review instrument); Multimedia Appendix 6	*Yes*
**11. Analysis**	a. Qualitative and quantitative methods used to draw inferences from the data. b. Methods for understanding variation within the data.	Section 2.5 (intervention coding); Section 2.6 (Likert scale + qualitative); Section 2.7	*Partial — No statistical tests (acknowledged as limitation due*

		(F-ACAD comparison)	*to N=2 reviewers and single-case design)*
**12. Ethical Considerations**	Ethical aspects of implementing and studying the intervention(s) and how they were addressed, including formal ethics review and potential conflict(s) of interest.	Section 2.9; Conflicts of Interest section	*Yes*
**Results — What did you find?**			
**13. Results**	a. Initial steps of the intervention(s) and their evolution over time. b. Details of the process measures and outcome. c. Contextual elements that interacted with the intervention(s). d. Observed associations between outcomes, interventions, and relevant contextual elements. e. Unintended consequences. f. Details about missing data.	Sections 3.1–3.7; Tables 1–5; Figures 2–4; Multimedia Appendices 4, 7–8	*Yes (a–d); N/A (e–f) — No unintended consequences observed; no missing data in this design*
**Discussion — What does it mean?**			
**14. Summary**	a. Key findings, including relevance to the rationale and specific aims. b. Particular strengths of the project.	Section 4.1	*Yes*
**15. Interpretation**	a. Nature of the association between the intervention(s) and the outcomes. b. Comparison of results with findings from other publications. c. Impact of the project on people and systems. d. Reasons for any differences between observed and anticipated outcomes. e. Costs and strategic trade-offs, including opportunity costs.	Sections 4.2.1–4.2.6	*Yes*
**16. Limitations**	a. Limits to the generalizability of the work. b. Factors that might have limited internal validity such as confounding, bias, or imprecision. c. Efforts made to minimize and adjust for limitations.	Section 4.3 (7 dedicated subsections)	*Yes*
**17. Conclusions**	a. Usefulness of the work. b. Sustainability. c. Potential for spread to other contexts. d. Implications for practice and for further study. e. Suggested next steps.	Section 4.4 (broader implications); Section 5 (Conclusion)	*Yes*
**Other Information**			
**18. Funding**	Sources of funding that supported this work. Role, if any, of the funding organization in the design, implementation, interpretation, and reporting.	Funding section	*Yes — No external funding received*
